# Anorexia Nervosa With Comorbid Severe Depression: A Systematic Scoping Review of Brain Stimulation Treatments

**DOI:** 10.1097/YCT.0000000000000922

**Published:** 2023-04-14

**Authors:** Peter Andersson, Esmail Jamshidi, Carl-Johan Ekman, Kristina Tedroff, Jonnie Björkander, Magnus Sjögren, Johan Lundberg, Jussi Jokinen, Adrian E. Desai Boström

**Affiliations:** From the ∗ Department of Clinical Neuroscience/Psychology, Karolinska Institute, Stockholm; † Center for Clinical Research Dalarna, Uppsala University, Falun; ‡ Stockholm Health Care Services, Region Stockholm, Stockholm; § Department of Clinical Sciences/Psychiatry, Umeå University, Umeå; ∥ Centre for Psychiatry Research, Department of Clinical Neuroscience, Karolinska Institutet, & Stockholm Health Care Services, Region Stockholm, Karolinska University Hospital, SE-171 76 Stockholm, Sweden; ¶ Stockholm Health Care Services, Region Stockholm, Karolinska University Hospital; # Department of Women's and Children's Health/Neuropediatrics, Karolinska Institute, Stockholm, Sweden

**Keywords:** severe anorexia nervosa, inpatients, ECT, DBS, MDD, treatment guidelines

## Abstract

Major depressive disorder (MDD) is highly prevalent in individuals with anorexia nervosa (AN) and is a predictor of greater clinical severity. However, there is a limited amount of evidence supporting the use of psychotropic medications for its management. A systematic scoping review was conducted to assess the current literature on brain stimulation treatments for AN with comorbid MDD, with a specific focus on MDD treatment response and weight restoration. This review was conducted according to Preferred Reporting Items for Systematic Reviews and Meta-Analyses guidelines, and the PubMed, PsycInfo, and MEDLINE databases were searched until July 2022 using specific key words related to AN and brain stimulation treatments. A total of 373 citations were identified, and 49 treatment studies that met the inclusion criteria were included in the review. The initial evidence suggests that electroconvulsive therapy, repetitive transcranial magnetic stimulation, and deep-brain stimulation may be effective in managing comorbid MDD in AN. Emerging evidence suggests that transcranial direct current stimulation may have a positive effect on body mass index in individuals with severe to extreme AN. However, there is a need for the development of better measurement techniques for assessing the severity of depression in the context of AN. Controlled trials that are adequately designed to account for these limitations are highly warranted for deep-brain stimulation, electroconvulsive therapy, and repetitive transcranial magnetic stimulation and hold promise for providing clinically meaningful results.

Anorexia nervosa (AN) is a severely debilitating psychiatric condition characterized by restrictive caloric intake, fear of weight gain, and distorted body perception.^1^ Disease onset typically occurs in adolescence, with a peak incidence at 13 to 18 years.^2,3^ Lifetime prevalence is estimated at 0.80% and females are significantly overrepresented.^4^ The *Diagnostic and Statistical Manual of Mental Disorders*, *Fifth Edition*, *Text Revision*, uses the body mass index (BMI) to divide AN into the following 4 subcategories of disease severity: mild (BMI >17 kg/m^2^), moderate (BMI 16–16.99 kg/m^2^), severe (BMI 15–15.99 kg/m^2^), and extreme (BMI < 15 kg/m^2^).^5^ Despite suggested improvements to inpatient care over time for AN,^6,7^ there is a high risk of relapse during the first year after discharge.^8^ Furthermore, a substantial proportion of AN-related treatment episodes in modern specialized inpatient units do not result in satisfactory outcomes.^9,10^ For example, in a 1-year follow-up study, Meule et al^11^ depicted inpatient treatment as highly effective for improving body weight and eating disorder symptoms—effects that appeared stable at endpoint according to self-reported BMI accounts. Nevertheless, the study demonstrated a high risk of relapse within the first year after discharge (consistent with the previous findings^8^) and indicated substantial individual differences in treatment response, pointing to the existence of distinct subgroups with unsatisfactory outcomes, and worsening of symptoms after discharge. Predictors of these negative outcomes include older age, longer duration of illness, and the occurrence of previous inpatient treatment episodes.^11^ Thus, there is a need not only to study specific AN subgroups exhibiting unsatisfactory outcomes after inpatient care but also to develop treatment methods for improving prognoses in both the short and long term for these disadvantaged patient groups.

Major depressive disorder (MDD) is highly prevalent in AN inpatients,^12^ constituting the strongest comorbid negative predictor of weight gain during AN treatment,^13^ as well as exacerbating risk of suicide, aphagia and pervasive refusal syndrome,^14^ and conferring substantial additive excess mortality.^15^ In addition, comorbid MDD in AN predicts higher clinical severity.^16^ Previous research explicitly states that sharing similar signs and symptoms, familial tendencies, and neuroendocrine abnormalities may make it difficult to clinically distinguish MDD and AN, which poses a risk of misdiagnosis.^17^ There are also studies that support the existence of clinically relevant associations between MDD symptoms and eating disorder psychopathology in inpatient treatment settings.^18^ Studies investigating AN inpatients indicate that comorbid depression is common.^12,16^ The long-term importance of addressing comorbidities in AN is underlined by the increased risk of long-term fatal outcomes conferred by the presence of concomitant diagnoses. The detrimental long-term effects of psychiatric comorbidity are borne out by studies indicating substantial increases in mortality.^15,19^ Mounting evidence implicates comorbid MDD as an especially important treatment target for achieving favorable outcomes in inpatient settings.^13,18,20^ The global negative influence of depression and anxiety disorders on eating disorder psychopathology in both men and women have been further substantiated in population-representative surveys (associations that were independent of BMI, age, and income).^21^ Further complicating the treatment of these patients is that weight gain in the early stages of AN inpatient treatment has been associated with exacerbation of depressive symptoms, which is believed to be an outcome conferred by effects of acute BMI increases on body dissatisfaction and weight/shape concerns. In contrast, after targeted and effective treatment of comorbid MDD, improvements in depressive and AN-specific symptoms and the increase in BMI followed a parallel course.^22^ Existing studies implicate substantial placebo-response rates in MDD and high natural course remittance rates in both adult^23^ and adolescent^24^ populations, which underlines the importance of adequate accounting for placebo-response rates and natural course remittances, before inference of any beneficial effect from active treatment.

Evidence supporting any psychotropic medication in management of the severe AN inpatient with comorbid MDD^25^ is scarce. Acute clinical conditions sometimes necessitate informing clinical practice based on observational studies in target populations, rather than randomized control trials (RCTs),^26^ and may provide heuristic information regarding the clinical management of this group.^27^ Emerging treatment studies, the majority of which are uncontrolled, provide preliminary support for noninvasive brain stimulation such as repetitive transcranial magnetic stimulation (rTMS), transcranial direct current stimulation (TDCS), electroconvulsive therapy (ECT), and invasive brain stimulation (deep brain stimulation [DBS] and vagus nerve stimulation [VNS]^28^) in the management of eating disorders and/or common comorbidities (MDD, anxiety disorders, etc). However, most of these studies primarily investigate peripheral outcome variables that are not directly associated with acute health concerns (ie, weight and/or depressive symptoms), rendering it a challenge to deduce their relevance to the comorbid MDD/severe AN inpatient.^29^ Duriez et al^29^ recently presented a review of brain simulation techniques in eating disorders; however, it did not pertain exclusively to AN patients and lacked essential details in relation to reported BMI and MDD outcomes (ie, rating scales used, magnitude of alterations in BMI and depressive symptoms, severity of AN and MDD, treatment duration). Thus, it seems that this field lacks a comprehensive scoping review investigating treatment trials of invasive and noninvasive brain stimulation in relation to severe AN with/without comorbid MDD and with a specific focus on effects on BMI and depressive symptoms. The objective of this study was to investigate whether BMI and MDD outcome measurements in relevant studies are rigorously reported, to highlight gaps in the literature, and to provide directions for future trials. A secondary objective was assessment of the overall merits of conducting further studies on the effects conferred by these regimens in the context of severe AN with comorbid MDD.

## METHODS

### Search Processes

This review was conducted according to PRISMA (Preferred Reporting Items for Systematic Reviews and Meta-Analyses) guidelines.^30^ Until July 2022, the PubMed, PsycInfo, and MEDLINE (OVID) databases were searched using the terms: (anorexia nervosa [Title/Abstract], OR anorexia [Title/Abstract], OR eating disorder [Title/Abstract], OR eating disorders [Title/Abstract]), AND (ECT [Title/Abstract], OR electroconvulsive therapy [Title/Abstract], OR electroshock therapy [Title/Abstract], OR electroshock [Title/Abstract], OR tDCS [Title/Abstract], OR transcranial direct current stimulation [Title/Abstract], OR rTMS [Title/Abstract], OR transcranial magnetic stimulation [Title/Abstract], OR DBS [Title/Abstract], OR deep brain stimulation [Title/Abstract] OR vagal nerve stimulation [Title/Abstract] OR VNS [Title/Abstract]). A total of 357 unique articles were identified using these search terms. Additional articles were identified by detecting similar articles and those with titles containing our search terms. Articles were selected by title and abstract; the entire article was read if the title/abstract concerned a studied brain stimulation treatment, AN, and MDD. References for the articles selected were also investigated to identify additional studies that met the inclusion criteria. The review protocol was created a priori but was not registered.

### Study Selection

Articles were included in the review according to the following inclusion criteria: English abstract, publication in peer reviewed journals, relevant brain stimulation treatment performed in humans with AN, and reporting on BMI/weight, or MDD outcomes (clinical assessments of MDD outcomes were included). Articles were excluded by title, abstract, or full text because of irrelevance to the topic in question. Further exclusion criteria were articles not written in the English language, unpublished dissertations and theses, and other nonpeer-reviewed material.

### Data Extraction

The search was individually performed by three members of the research team (P.A., E.J., and A.D.B.). All articles published in English up until July 2022 were retrieved. A total of 357 retrieved articles were independently reviewed and selected based on the inclusion and exclusion criteria. There were 16 additional studies identified that met the criteria for inclusion through checks of the references for selected articles, resulting in 373 articles (357 + 16). The authors subsequently re-evaluated the results, with presentation of only salient results. After the literature re-evaluation, P.A. and A.D.B. individually scrutinized all retrieved articles, followed by the manual extraction of data for treatment outcomes pertaining to weight gain and depressive symptoms (see Supplemental Table 1, Supplemental Digital Content 1, http://links.lww.com/JECT/A189). For weight, BMI was the preferred reporting format, but where studies only reported weight changes in kilograms or pounds, these numbers were retrieved. For depressive symptoms, data pertaining to rating scales measuring depressive symptoms were preferentially extracted, but for articles in which severity of depressive symptoms was exclusively described qualitatively, these descriptions were retrieved. The data extraction process included a classification of the severity of AN and depressive symptoms at baseline in the samples included. Classification of severity of AN was based on reported pretreatment BMI values, which were classified according to the following 4 *Diagnostic and Statistical Manual of Mental Disorders*, *Fifth Edition*, *Text Revision*, subcategories: mild (BMI > 17 kg/m^2^), moderate (BMI 16–16.99 kg/m^2^), severe (BMI 15–15.99 kg/m^2^), and extreme (BMI < 15 kg/m^2^). The classification of baseline depressive symptoms was based on reported values on rating scales measuring depressive symptoms and classified according to established cutoff points for each respective scale (see Supplemental Notes, http://links.lww.com/JECT/A190). Clinical assessments of MDD severity before, during, or after treatment, were qualitatively interpreted and summarized. Data regarding treatment duration and, when available, psychiatric comorbidities, were extracted. Any disagreement regarding the severity classification was resolved by consensus discussion. The data extracted by P.A. and A.D.B. were scrutinized for inconsistencies by E.J., who scrutinized such articles in their entirety, and extracted the data concerned. Finally, any inconsistencies past this point were resolved through consensus discussions among the 3 authors and finalized by majority vote. The following information was extracted from the included studies. (*a*) Publication data: Identification data such as the authors' names and publication dates, as well as details on the study designs. This information is important to understand the context and methodology of the studies. (*b*) Demographics: Information on the number of participants diagnosed with AN, their subtype of AN (restrictive, restrictive/purging, binge/purging, or unspecified), the setting of the study (inpatient, outpatient, or mixed), and the age and gender of the participants. This information allows for understanding the characteristics of the population being studied. (*c*) Clinical variables: This category includes information pertaining to treatment and outcomes, such as the number of sessions, electrode placement, treatment frequency, and total duration of the study, as well as information on the presence of any psychiatric comorbidities, BMI/weight data, including baseline BMI and inferred AN severity at baseline, end of treatment or at follow-up, and information on MDD including the outcome measurement instrument, mean score and standard deviation at baseline, inferred MDD severity at baseline, as well as mean score and standard deviation at follow-up or end of treatment. This information provides insight into the clinical aspects of the disorder and the effectiveness of different treatments.

## RESULTS

### Study Characteristics

**FIGURE 1:**
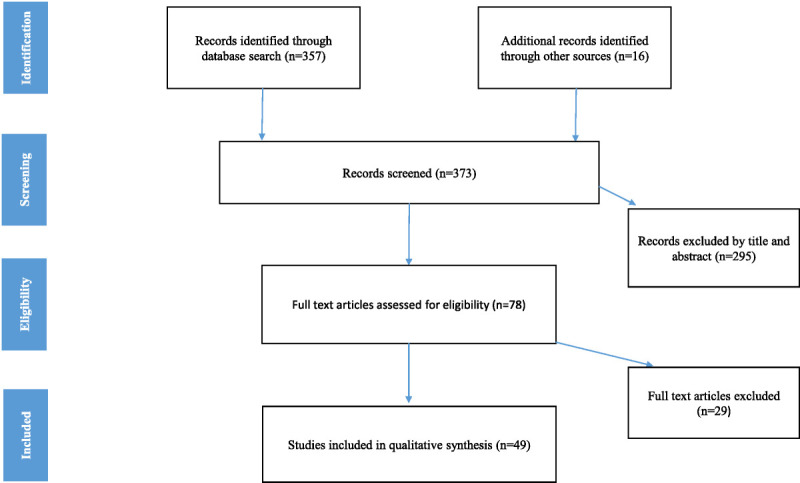
Study flow diagram. The results of the search and the process of screening and selecting studies for inclusion in the review.

A flowchart of articles selected for the review is provided in Figure 1. The PubMed database search provided a total of 357 citations, with 16 additional studies identified through other sources. Thus, a total of 373 studies were screened. There were 295 records excluded after title and abstract screening, resulting in 78 full texts that were assessed for eligibility. After this assessment, 29 articles were excluded, resulting in the inclusion of 49 studies in the final qualitative synthesis (rTMS [n = 13], tDCS [n = 4], ECT [n = 14], DBS [n = 18], VNS [n = 0]). It should be noted that some overlap could exist between samples in the included studies. Grounds for exclusion included lack of relevance to the general topic and studies describing brain stimulation treatment in eating disorders that did not include AN subjects.

### Repetitive Transcranial Magnetic Stimulation

We identified 163 patients, from 18 to 52 years of age with AN who underwent rTMS as part of their treatment (excluding subjects receiving sham treatment). A summary of extracted data is presented in Supplemental Table 1 (Supplemental Digital Content 1, http://links.lww.com/JECT/A189). Patients underwent between one to 42 sessions, with a mean of 20.5 treatments. Cases of explicitly detailed comorbid psychiatric diagnoses included MDD (n = 20), bipolar disorder (n = 8), social anxiety (n = 3), borderline personality disorder (n = 1), panic disorder (n = 1), obsessive compulsive disorder (OCD, n = 7), unspecified anxiety disorder (n = 1), and posttraumatic stress disorder (PTSD, n = 14). Major depressive disorder severity was only explicitly stated in one case. Based on evaluations of depression rating scales, a total of 31 individuals were indicated as severe MDD (ie, 30 inferred as severe, and one explicitly stated as such). Of 13 articles, 8 included patients who met criteria for severe AN at baseline, including 2 RCTs. Only female subjects were included. In the 2 RCTs reporting on BMI and severe AN, the mean increase in BMI after treatment was 0.33 kg/m^2^, and neither RCT could evince superiority over placebo in achieving weight gain.^31,32^ Most patients with inferred or reported severe MDD showed considerable improvement after a course of rTMS, conferring a mean reduction of 9.6 points (constituting a change from “severe” to “moderate” depression) in the Depression Anxiety Stress Scales 21 (DASS-21)—an effect indicated as markedly superior to placebo across the 2 RCTs.^31,32^ Notably, these cases related to a majority of severe AN patients. It should be noted that these findings are limited, in that the psychometric properties of the DASS-21 rating scale have not been extensively investigated for adequate measurement of depressive symptoms in the context of clinical AN. For example, previous studies implicated that the DASS-21 lacks consistently substantiated abilities for discriminating between MDD and anxiety disorders (DASS-21) in psychiatric patients and the general public.^33^ This represents an important source of potential confound given the high prevalence of comorbid anxiety disorders in AN.^34^ Four observational studies reported on MDD outcomes, with mixed results (ie, three not reporting any improvement in cases inferred as exhibiting mild/moderate MDD at baseline,^35–37^ and one observing a mean 18-point reduction on the HAM-D in a case report of a 24-year-old woman with severe MDD^38^). The left dorsolateral prefrontal cortex (DLPFC) was the treatment target in nine of the 13 included studies, while 2 studies targeted the dorsomedial prefrontal cortex (DMPFC), and one study described deep transcranial stimulation of the insula. In one DLPFC-targeted study, laterality was not specified, Supplemental Table 1 (Supplemental Digital Content 1, http://links.lww.com/JECT/A189). Regarding the 2 RCTs measuring BMI in severe AN (pertaining to the same group of patients but reporting at different time points for follow-up), both studies investigated the effects of 20× neuronavigated high-frequency stimulation (10) Hz of the left DLPFC, administered in 20 × 5-second trains with 55-second intertrain intervals, for a total of 1000 pulses during each 20-minute treatment session.^31,32^ Dunlop et al^39^ investigated rTMS targeting the DMPFC and delivered at 10 Hz and 120% of motor threshold, in pulses of 5 seconds on and 10 seconds off, for a total of 3000 pulses per hemisphere, with left then right lateralized coil orientation. Woodside et al^40^ studied a sample of mixed eating disordered patients (total n = 14, six of which were AN participants) who were administered DMPFC-targeted rTMS with three treatment regimens—a 20-Hz stimulation regimen, theta-burst stimulation, and the above-detailed 10-Hz regimen previously implemented by Dunlop et al.^39^ Knyahnytska et al^37^ studied a novel approach in which a so-called H-coil was used to attempt deep transcranial magnetic stimulation of the insula. This was delivered at a frequency of 18 Hz, with 36 pulses of 2 seconds on and 20 seconds off during 80 trains, for a full duration of 20 minutes per session (42 sessions per subject).^37^

### Electroconvulsive Therapy

There were 46 AN patients identified, aged from 12 to 94 years, who underwent ECT as part of their treatment. Extracted data are summarized in Supplemental Table 1 (Supplemental Digital Content 1, http://links.lww.com/JECT/A189). Patients underwent between 5 and 31 sessions, with a mean of 16.4 treatments. Laterality was reported as bitemporal/bilateral in all cases but two^41,42^ for which electrode placement was reported. Psychiatric comorbidities included 40 MDD cases (of which 32 were indicated as severe), schizophrenia (n = 2), nonsuicidal self-injury (NSSI, n = 29), generalized anxiety disorder (n = 1), OCD (n = 12), unspecified anxiety and personality disorders (n = 7 and n = 5, respectively), and PTSD (n = 6). Notably, only 3 of 46 patients fulfilled criteria for severe AN at baseline. Of the 46 patients, 45 were female. Of 2 cases reporting on BMI and severe AN, the median increase in BMI after treatment was 0.4 kg/m^2^, and in one article reporting weight, the weight gain after completion of treatment was 4 kg. Most patients with inferred severe MDD showed considerable improvement after a course of ECT, demonstrating a 50% reduction in the Montgomery-Åsberg Depression Rating Scale (MADRS),^43^ or a 2-point average in reductions in the Clinical Global Impressions Scale (CGI-S)^44^ (from “severely ill” to “moderately ill”). Of significance is that these cases were related to most patients with mild AN.

### Transcranial Direct Current Stimulation

There is a relative paucity of treatment studies using transcranial direct current stimulation in AN. Duriez et al^29^ included results from 2 smaller (n = 10, n = 7) open-label studies. The literature review conducted for this study identified 2 additional randomized controlled trials (RCTs) (Supplemental Table 1, Supplemental Digital Content 1, http://links.lww.com/JECT/A189)^45,46^ that report conflicting results. In the first RCT, Costanzo et al^46^ assigned 23 adolescents (22 females, 1 male) with severe to extreme AN to either 18 sessions of left anodal/right cathodal prefrontal cortex tDCS or family-based therapy. The study found a statistically significant increase in mean BMI after the 6-week tDCS treatment (mean increase: approximately 1.9 kg/m^2^), compared with a smaller increase in the control group (mean increase: approximately 0.6 kg/m^2^). In addition, the tDCS group also exhibited slightly improved reductions in self-rated assessments on the Children's Depression Inventory (ie, a mean reduction of 11.8 points in the tDCS group compared with 7.6 in the active control group). The second RCT, conducted by Bauman et al,^45^ was a double-blind, controlled trial in 43 female inpatients with moderate to severe AN, randomized to receive either 10 sessions of anodal tDCS treatment over the left DLPFC or sham tDCS. The study found that BMI values were marginally improved in both groups 4 weeks after treatment, but the authors did not provide details on BMI at follow-up. In addition, the study reported that the sham group had greater reductions in depression scores 4 weeks after treatment, but the data were not provided. Neither of the 2 open-label studies measured BMI at follow-up, which precludes any conclusions from these reports regarding the potential effects of tDCS on weight gain. However, both studies reported on depressive symptoms at baseline and posttreatment. After 10 sessions of tDCS, Khedr et al^47^ observed marginal improvement on the Beck Depression Inventory II (BDI-II) in 6 of 7 patients assessed after treatment, with 3 of the 7 patients progressing to present with improvements at the 1-month follow-up.^47^ Similarly, Strumila et al^48^ reported on improvements in the BDI^49^ after 20 times tDCS sessions (posttreatment), and at 1-month follow-up, with a moderate effect size of 0.47. Both studies targeted the left DLPFC with 2-mA anodal tDCS. In the study by Khedr et al,^47^ this was administered once daily for 25 minutes at a frequency of 5 sessions per week, while Strumila et al^48^ studied a treatment regimen comprising 2 daily 25-minute sessions, administered Monday to Sunday over 2 weeks.

### Deep Brain Stimulation

There were 118 patients identified between the ages of 16 to 60 years with AN who underwent DBS as part of their treatment. Extracted data are summarized in Supplemental Table 1 (Supplemental Digital Content 1, http://links.lww.com/JECT/A189). Outcomes were assessed between 1 and 50 months, with a mean duration of 16.6 months. Psychiatric comorbidities included MDD (n = 103, of which 23 were indicated as severe). Other comorbidities included OCD (n = 35), PTSD (n = 26), generalized anxiety disorder (n = 9), panic disorder (n = 3), unspecified anxiety disorder (n = 5), and substance abuse (n = 2). Notably, 85 of 118 patients met criteria for severe or extreme AN at baseline or belonged to samples for which the mean was below this threshold. Among the 118 patients, 115 were reported as female and 2 were male, with the gender of one patient not being reported. Of the articles reporting on BMI and severe/extreme AN, the median increase in BMI after treatment at last measurement point was 4.72. After treatment, 57 of 118 patients had BMI > 17.5 kg/m^2^ or belonged to samples for which mean BMI was >17.5 kg/m^2^ as compared with before treatment. Most patients showed considerable improvement after the course of DBS, with improvement in depressive symptoms either directly reported or suggested, in sample means for 80 patients. Of note is that at the time of treatment with DBS, all of these patients were critically ill, and most had failed multiple treatments before consideration and initiation of treatment. With regard to MDD, 72 patients had a reported MDD outcome at baseline and follow-up and were depressed at baseline (irrespective of severity). Of these, 64 reported improvements in depression severity at the latest measurement point (follow-up times varied between 3 months and 2 years after intervention). There was a mean reduction of 10.8 points observed in the Hamilton Depression Rating Scale (whereby at least moderate severity MDD could be inferred), indicating remission in 21 cases in which baseline results pointed to at least moderate-severity MDD. Comorbid severe MDD was inferred or reported in 23 of these cases, for which there was a substantial improvement in depressive symptoms. For example, at end point, on average, both the MADRS and the BDI were reduced by 30 points (from “severe” to “mild,” n = 1), and 18 points (from “severe” to “moderate,” n = 22). In the 18 included studies, 7 included subjects administered DBS targeting the nucleus accumbens, 4 studies described subcallosal cingulate stimulation, 3 described stimulation of the bed nucleus of the stria terminalis, and other studied a description of stimulation of each of the subgenual cingulate cortex, anterior limbs of the internal capsule, ventral capsule/ventral striatum, genu of the corpus callosum, and/or medial forebrain bundle in the posterior hypothalamic region (some studies included several subjects who received DBS at differing locations) (Supplemental Table 1, Supplemental Digital Content 1, http://links.lww.com/JECT/A189).

## DISCUSSION

This scoping review of brain stimulation treatments in the context of AN with comorbid MDD has included 49 treatment studies covering rTMS, tDCS, ECT, VNS, and DBS. (1) For rTMS, most patients with inferred or reported severe MDD showed considerable improvement after a course of rTMS, conferring a mean reduction of 9.6 points in the DASS-21 (constituting a change from “severe” to “moderate” depression)—an effect noted as markedly superior to placebo across the 2 RCTs. However, caution is warranted when inferring MDD-specific effects of rTMS in the context of AN based on these studies. Notably, the psychometric properties of the DASS-21 rating scale have not been extensively investigated for adequate measurement of depressive symptoms in the context of clinical AN. For example, previous studies implicated that the DASS-21 lacks consistently substantiated abilities for discriminating between MDD and anxiety disorders (DASS-21) in psychiatric patients and the general public.^33^ This represents an important source of potential confound, given the high prevalence of comorbid anxiety disorders in AN.^34^ In conclusion, preliminary results indicate that an average of 20.5 rTMS treatments—while associated with nonmeaningful effects on BMI—may confer reductions on the DASS-21 rating scale. However, causal inferences regarding the utility of this treatment modality are diminished by the small number of studies and the use of unsubstantiated tools for measurement of depressive symptoms. (2) The studies included in this review consisted of 2 RCTs and 2 open-label studies, all of which investigated the use of tDCS in individuals with AN. One RCT, which recruited primarily female adolescents with severe to extreme AN (n = 23), found that 18 sessions of tDCS led to superior improvements in BMI compared with an active placebo with family-based therapy. In contrast, the other RCT, which included 43 female inpatients with moderate AN, did not observe any improvements in BMI after 10 sessions of tDCS compared with sham-tDCS treatment. Neither of these RCTs observed any effects on reducing depressive symptoms. These findings suggest emerging support for tDCS treatment in improving BMI in females with severe to extremely severe AN, but not having any effect on symptoms of MDD and not improving BMI in milder cases of AN. However, these findings contrast with those of 2 smaller, noncontrolled studies that observed moderate improvement in depressive symptoms assessed using the BDI-II but neither of these studies included BMI measurement at follow-up. The potential for publication bias and unaccountable confounders such as placebo response rates and natural course remittance reduce confidence in these observations. (3) Regarding ECT, most patients with inferred severe MDD and AN showed considerable improvement after a course of ECT, demonstrating a 50% reduction in the MADRS, or an average reduction of 2 points in the CGI-S (from “severely ill” to “moderately ill”). Notably, these cases related to a majority of patients with mild AN. Overall, preliminary results indicate that an average of 16 bilateral ECT treatments—while associated with nonmeaningful clinical improvements in BMI—may bestow substantial reductions on both MADRS and CGI-S scores. However, it would appear as if the small number of studies, putative publication bias, and lack of well-designed RCTs to adequately account for placebo-response rates and natural course-remittances, weaken any causal inference regarding the utility of this treatment modality. (4) The literature review conducted for this study did not identify any published clinical studies that investigate the use of VNS in patients with AN and comorbid MDD who have not recovered. As a result, this study was unable to assess the feasibility and clinical utility of VNS in this population. However, it is worth noting that a clinical trial is currently recruiting subjects to investigate the effects of noninvasive VNS for the treatment of low weight eating disorders in adolescents. The study record can be found on ClinicalTrials.gov under the identifier NCT05554172.^50^ (5) In the case of DBS, observational data support DBS as a potential treatment to achieve long-term weight gain and reduce depressive symptoms in severe AN with comorbid MDD. However, in particular, the absence of control treatment trials should be noted—not least considering the long average follow-up time, which could indicate that in some cases, improvement in natural disease course could account for parts of these improvements. In conclusion, results from primarily observational level research suggest that rTMS, ECT, and DBS may confer positive effects on depression severity as adjunctive treatment in samples with AN and comorbid MDD. The low quality of available data precludes any definite conclusions regarding the effectiveness of these brain stimulation techniques in management of the target population. Notably, interpretation is complicated by the weak evidence in support of commonly used outcome variables to assess MDD severity in AN. Importantly, the outcome of clinical value of brain stimulation techniques on managing MDD in the context of AN cannot be reliably measured in this population using measurement instruments with unsubstantiated psychometric properties. Development of better measurement techniques for depression severity in the context of AN is an urgent requirement. Controlled trials adequately designed to account for such limitations are highly warranted with regard to rTMS, ECT and DBS.

### Contribution to Weight Normalization

The primary objective of treating severely malnourished individuals with AN is to achieve weight stabilization, and there is evidence that both DBS and tDCS may provide positive outcomes. While a small RCT suggests that tDCS may improve BMI in individuals with severe to extreme AN, no such effect has been observed in less severe cases. Further replication of these findings in different patient populations is necessary to confirm causality. The effects of DBS have been reported in studies with extended follow-ups that lack control groups, raising questions about whether the observed improvements are treatment related or simply because of the natural course of the disorder. Nevertheless, the magnitude of improvement seen in severely affected AN patients participating in DBS trials is unlikely to occur spontaneously, which tentatively supports the idea that DBS may have positive long-term effects on weight gain in this population. It should be noted that the effects of DBS are not consistent and depend on the specific brain circuitry targeted, which has varied significantly across studies.

### Managing Key Knowledge Gaps

The first important issue that should be addressed is the measurement of depressive symptoms in the context of AN. There was considerable heterogeneity in the reported measurement instruments used to assess depression severity across all studied treatment modalities. Items used include self-administered questionnaires, and rating scales based on semistructured interviews. The validity of self-rating scales has been subject to extensive debate, partly because of poor concordance with clinician-rated scales.^51–54^ Hence, direct comparisons between studies relying on self-reported scales with those depending on clinician-rated scales could be misguided. Moreover, a study by Dêbska et al^55^ underlined the complexity of diagnosing MDD in patients diagnosed with AN, noting the secondary nature of depressive symptoms in some patients experiencing AN, and suggesting that results of the BDI need to be confronted with the clinical picture, to arrive at the correct diagnosis. None of the reported rating scales have been extensively validated for measurement of MDD severity in the context of clinical AN. Furthermore, some of these rating scales have unsubstantiated psychometric properties for measuring MDD in non-AN populations Children's Depression Rating Scale-Revised,^56^ or lack consistently substantiated adequate abilities for discrimination between MDD and anxiety disorders (DASS-21) in psychiatric patients and the general public.^33^ The second important issue is that causality cannot be inferred from these studies, the majority of which were observational. One possible suggestion for research going forward—aside from RCT initiatives—could be controlled studies with clearly predefined rules for stopping for benefit and other safety protocols and overseen by independent data review committees.^27,57^ More well-designed and preregistered observational studies using accurate outcome measures could also be beneficial.^58^ From this perspective, psychometric research focused on measurement of comorbid MDD in AN could be of utility for the standardization of measurement methods in research and clinical practice, allowing for increased comparability across samples. A summary of knowledge gaps and research questions identified throughout the review process is presented in Table 1.

**TABLE 1:** Prioritized Research Questions and Knowledge Gaps for Future Studies on Brain Stimulation Treatments in AN With Comorbid MDD

Subject	Knowledge Gap	Research Questions
Illness severity	Studies included in the analysis infrequently report on the symptom severity of MDD and/or AN, and the subject population encompasses the entire spectrum of severity levels (ie, mild, moderate, severe, and extremely severe) for both conditions	Can it be definitively concluded that the severity of symptoms in MDD and/or AN do not impact the outcomes of brain stimulation treatments? Can findings from the treatment of mild to moderate MDD and/or AN patients be reliably applied to patients with severe or extreme symptom severity, or is this analogous to comparing apples to oranges?
Causality	93% of studies are observational (causality not investigated) Several studies reported on subjects simultaneously receiving other treatments (ie, nasogastral tube feeding, psychotherapy and/or pharmacotherapy)	Can it be definitively determined that the observed treatment outcomes accurately reflect true efficacy? Are the study design and methodology robust enough to account for publication bias, placebo response, and spontaneous remission? Are the studies designed to specifically examine the effects of brain stimulation treatments, or could the influence of concurrent treatments confound the results?
Quality of reporting	The observational studies included were not prospectively registered and did not conform to established reporting guidelines.	Does the study provide the author with a clear presentation of the work and provide the reader with appropriate information to enable critical appraisal of the research? Were primary and secondary outcome variables determined a priori and rigorously adhered to? Did the study adhere to a recognized reporting guideline for observational studies?
Confound from measurement instruments to assess MDD in context of AN	60% of studies reported on any MDD outcome (*a*) 12.5% pertained exclusively to self-rated rating scales (*b*) 22.5% exclusively to clinician-rated scales (*c*) 17.5% included both clinician and self-rated scales (*d*) 12.5% pertained exclusively to descriptions of clinical assessments (ie, no MDD-specific rating scale)	Evaluating treatments for MDD in the presence of AN requires reliable measures of depression severity. The use of measurement instruments with inadequate psychometric properties is not sufficient. There is a pressing need for improved methods of assessing depression severity in AN. Until such instruments are developed, a combination of clinician-rated instruments with established psychometric properties and CGI-S assessments by 2 independent expert raters may enhance the reliability of reported outcomes.
Sex, gender and gender identity	No studies addressing the role of gender identity >95% of participants female	Do different subtypes of MDD display disparities in prevalence across genders? Is there a correlation between gender identity and MDD in AN? Do gender or sex differences exist in the outcomes of MDD in AN?
Age	(1) 90% of rTMS participants were 20–40-year-olds (2) Most ECT participants were in adolescence or young adulthood, but included a 94-year-old (3) tDCS participants were in young adulthood (4) DBS studies included both participants in young adulthood and 30–60-year-olds	Do differences in age affect the outcomes of MDD in AN from brain stimulation treatments?
Year of publication	All DBS, rTMS and tDCS studies were published between 2008–2020 50% of ECT studies were published in 2011–2021, 14% in 2001–2010, 14% in 1990–2000 and 22% before 1990	Are brain stimulation treatments comparable between the pre- and post-2000 era? Have the treatment instruments undergone substantial changes over the course of the studies?
Psychiatric comorbidities	Most studies reported on psychiatric comorbidities, including OCD, PTSD, and anxiety disorders	In the absence of validated measures to differentiate between MDD and comorbid anxiety disorders, both of which are prevalent in AN, can it be accurately concluded that reported outcomes solely reflect improvements in depression severity and not in anxiety disorders?
Demographics	(1) 50% of the rTMS studies were in the UK (2) 33% of the DBS studies were in Canada (3) 50% of the ECT studies were in the US	Do cultural variations exist in the response of AN patients with comorbid MDD to brain stimulation treatments?
Self-harm	Despite its high prevalence in AN, NSSI was only reported in 5% of the studies included.	Is the reporting of NSSI symptoms adequate? Does the study sample accurately reflect the condition being investigated?

### Limitations

The majority of the articles included were observational. Furthermore, caution is advised in terms of generalizing results to populations with less severe AN with comorbid MDD, for which psychosocial and psychological treatment options are currently recommended by international guidelines. Nevertheless, ECT is widely recommended, and highly effective, in the treatment of treatment refractory severe (or psychotic) MDD—and should not be disregarded as a treatment option in cases of severe AN with comorbid MDD unresponsive to other treatments.

## CONCLUSIONS

Preliminary evidence, primarily obtained from rating scales with questionable psychometric properties for measuring depression severity in individuals with AN and comorbid MDD, suggests that ECT, rTMS, and DBS may be effective in managing comorbid MDD in AN. However, these interventions seem to have limited impact on weight gain, with the exception of DBS, for which long-term observational data suggest at least some meaningful improvement. There is also emerging evidence to support the use of tDCS to improve BMI in females with severe to extremely severe AN, but it does not seem to reduce symptoms of MDD and may not be effective in milder cases of AN. No studies were identified on the use of VNS in nonrecovered AN patients with comorbid MDD, although a clinical trial has been registered. The measurement of the outcomes of clinical value of brain stimulation techniques on managing MDD in the context of AN is hindered by the use of measurement instruments with questionable psychometric properties. There is a pressing need for the development of better measurement techniques for depression severity in the context of AN. Controlled trials with designs that adequately account for these limitations are urgently needed for rTMS, ECT, and DBS and hold promise for providing clinically meaningful results.

## Supplementary Material

## References

[1] First MB Yousif LH Clarke DE, . *DSM-5-TR*: overview of what's new and what's changed. *World Psychiatry*. 2022;21:218–219. 35524596 10.1002/wps.20989 PMC9077590

[2] Campbell K Peebles R. Eating disorders in children and adolescents: state of the art review. *Pediatrics*. 2014;134:582–592. 25157017 10.1542/peds.2014-0194

[3] Kappou K Ntougia M Kourtesi A, . Neuroimaging findings in adolescents and young adults with anorexia nervosa: a systematic review. *Children*. 2021;8, 137. doi:10.3390/CHILDREN8020137. 33673193 PMC7918703

[4] Udo T Grilo CM. Prevalence and correlates of *DSM-5*-defined eating disorders in a nationally representative sample of U.S. adults. *Biol Psychiatry*. 2018;84:345–354. 29859631 10.1016/j.biopsych.2018.03.014 PMC6097933

[5] *Diagnostic and Statistical Manual of Mental Disorders* (5th Ed., Text Rev.) Arlington, Virginia: American Psychiatric Association, 2022 doi:10.1176/appi.books.9780890425787.

[6] Haas V Kohn M Körner T, . Practice-based evidence and clinical guidance to support accelerated re-nutrition of patients with anorexia nervosa. *J Am Acad Child Adolesc Psychiatry*. 2021;60:555–561. 32998025 10.1016/j.jaac.2020.09.010 PMC10863999

[7] Lindblad F Lindberg L Hjern A. Improved survival in adolescent patients with anorexia nervosa: a comparison of two Swedish national cohorts of female inpatients. *Am J Psychiatry*. 2006;163:1433–1435. 16877658 10.1176/ajp.2006.163.8.1433

[8] Berends T Boonstra N Van Elburg A. Relapse in anorexia nervosa: a systematic review and meta-analysis. *Curr Opin Psychiatry*. 2018;31:445–455. 30113325 10.1097/YCO.0000000000000453

[9] Wales J Brewin N Cashmore R, . Predictors of positive treatment outcome in people with anorexia nervosa treated in a specialized inpatient unit: the role of early response to treatment. *Eur Eat Disord Rev*. 2016;24:417–424. 27045727 10.1002/erv.2443

[10] Herpertz-Dahlmann B Schwarte R Krei M, . Day-patient treatment after short inpatient care versus continued inpatient treatment in adolescents with anorexia nervosa (ANDI): a multicentre, randomised, open-label, non-inferiority trial. *Lancet (London, England)*. 2014;383:1222–1229. 24439238 10.1016/S0140-6736(13)62411-3

[11] Meule A Schrambke D Furst Loredo A, . Inpatient treatment of anorexia nervosa in adolescents: a 1-year follow-up study. *Eur Eat Disord Rev*. 2021;29:165–177. 33230832 10.1002/erv.2808

[12] Blinder BJ Cumella EJ Sanathara VA. Psychiatric comorbidities of female inpatients with eating disorders. *Psychosom Med*. 2006;68:454–462. 16738079 10.1097/01.psy.0000221254.77675.f5

[13] Eskild-Jensen M Støving RK Flindt CF, . Comorbid depression as a negative predictor of weight gain during treatment of anorexia nervosa: a systematic scoping review. *Eur Eat Disord Rev*. 2020;28:605–619. 32886423 10.1002/erv.2787

[14] Carretier E Blanchet C Moro MR, . Comorbid major depressive disorder of anorexia nervosa in adolescence: a scoping review of treatment strategies. *Encephale*. 2021;47:72–78. 32933763 10.1016/j.encep.2020.05.017

[15] Kask J Ekselius L Brandt L, . Mortality in women with anorexia nervosa: the role of comorbid psychiatric disorders. *Psychosom Med*. 2016;78:910–919. 27136502 10.1097/PSY.0000000000000342

[16] Riquin E Raynal A Mattar L, . Is the severity of the clinical expression of anorexia nervosa influenced by an anxiety, depressive, or obsessive-compulsive comorbidity over a lifetime? *Front Psychiatry*. 2021;12:793. 10.3389/fpsyt.2021.658416 PMC8280337 34279519

[17] Garfinkel PE Garner DM Kaplan AS, . Differential diagnosis of emotional disorders that cause weight loss. *Can Med Assoc J*. 1983;129:939. 6367916 PMC1875814

[18] Sjögren M Støving RK. Anorexia nervosa: reduction in depression during inpatient treatment is closely related to reduction in eating disorder psychopathology. *J Pers Med*. 2022;12:682. 35629105 10.3390/jpm12050682 PMC9145215

[19] Kask J Ramklint M Kolia N, . Anorexia nervosa in males: excess mortality and psychiatric co-morbidity in 609 Swedish in-patients. *Psychol Med*. 2017;47:1489–1499. 28162109 10.1017/S0033291717000034

[20] Panero M Marzola E Tamarin T, . Comparison between inpatients with anorexia nervosa with and without major depressive disorder: Clinical characteristics and outcome. *Psychiatry Res*. 2021;297:113734. 33486276 10.1016/j.psychres.2021.113734

[21] Ernst M Werner AM Tibubos AN, . Gender-dependent associations of anxiety and depression symptoms with eating disorder psychopathology in a representative population sample. *Front Psychiatry*. 2021;12:645654. 33716837 10.3389/fpsyt.2021.645654 PMC7952511

[22] Shilton T Enoch-Levy A Giron Y, . A retrospective case series of electroconvulsive therapy in the management of comorbid depression and anorexia nervosa. *Int J Eat Disord*. 2020;53:210–218. 31639233 10.1002/eat.23181

[23] Timothy Walsh B Seidman SN Sysko R, . Placebo response in studies of major depression: variable, substantial, and growing. *JAMA*. 2002;287:1840–1847. 11939870 10.1001/jama.287.14.1840

[24] Thapar A Collishaw S Pine DS, . Depression in adolescence. *Lancet (London, England)*. 2012;379:1056–1067. 22305766 10.1016/S0140-6736(11)60871-4 PMC3488279

[25] Cassioli E Sensi C Mannucci E, . Pharmacological treatment of acute-phase anorexia nervosa: evidence from randomized controlled trials. *J Psychopharmacol*. 2020;34:864–873. 32448045 10.1177/0269881120920453

[26] Yeh RW Valsdottir LR Yeh MW, . Parachute use to prevent death and major trauma when jumping from aircraft: randomized controlled trial. *BMJ*. 2018;363:k5094. 30545967 10.1136/bmj.k5094 PMC6298200

[27] Paneth N Joyner M. The use of observational research to inform clinical practice. *J Clin Invest*. 2021;131. doi:10.1172/JCI146392. PMC7810491 33270605

[28] Kamel LY Xiong W Gott BM, . Vagus nerve stimulation: an update on a novel treatment for treatment-resistant depression. *J Neurol Sci*. 2022;434:120171. 35158102 10.1016/j.jns.2022.120171

[29] Duriez P Khalil RB Chamoun Y, . Brain stimulation in eating disorders: state of the art and future perspectives. *J Clin Med*. 2020;9:2358. 32717984 10.3390/jcm9082358 PMC7465000

[30] Moher D Liberati A Tetzlaff J, . Preferred reporting items for systematic reviews and meta-analyses: the PRISMA statement. *BMJ*. 2009;339:332–336. PMC3090117 21603045

[31] Dalton B Bartholdy S McClelland J, . Randomised controlled feasibility trial of real versus sham repetitive transcranial magnetic stimulation treatment in adults with severe and enduring anorexia nervosa: the TIARA study. *BMJ Open*. 2018;8:e021531. 10.1136/bmjopen-2018-021531 PMC6082449 30012789

[32] Dalton B Foerde K Bartholdy S, . The effect of repetitive transcranial magnetic stimulation on food choice-related self-control in patients with severe, enduring anorexia nervosa. *Int J Eat Disord*. 2020;53:1326–1336. 32309882 10.1002/eat.23267

[33] Ali AM Alkhamees AA Hori H, . The Depression Anxiety Stress Scale 21: development and validation of the Depression Anxiety Stress Scale 8-item in psychiatric patients and the general public for easier mental health measurement in a post COVID-19 world. *Int J Environ Res Public Health*. 2021;18, 10142. doi:10.3390/IJERPH181910142. 34639443 PMC8507889

[34] Swinbourne J Hunt C Abbott M, . The comorbidity between eating disorders and anxiety disorders: prevalence in an eating disorder sample and anxiety disorder sample. *Aust N Z J Psychiatry*. 2012;46:118–131. 22311528 10.1177/0004867411432071

[35] McClelland J Bozhilova N Nestler S, . Improvements in symptoms following neuronavigated repetitive transcranial magnetic stimulation (rTMS) in severe and enduring anorexia nervosa: findings from two case studies. *Eur Eat Disord Rev*. 2013;21:500–506. 24155247 10.1002/erv.2266

[36] Jaššová K Albrecht J Papežová H, . Repetitive transcranial magnetic stimulation (rTMS) treatment of depression and anxiety in a patient with anorexia nervosa. *Med Sci Monit*. 2018;24:5279–5281. 30057403 10.12659/MSM.908250 PMC6080581

[37] Knyahnytska YO Blumberger DM Daskalakis ZJ, . Insula H-coil deep transcranial magnetic stimulation in severe and enduring anorexia nervosa (SE-AN): a pilot study. *Neuropsychiatr Dis Treat*. 2019;15:2247. 31496707 10.2147/NDT.S207630 PMC6689531

[38] Kamolz S Richter MM Schmidtke A, . Transkranielle magnetstimulation gegen komorbide depression bei anorexie. *Nervenarzt*. 2008;79:1071–1073. 18661116 10.1007/s00115-008-2537-8

[39] Dunlop K Woodside B Lam E, . Increases in frontostriatal connectivity are associated with response to dorsomedial repetitive transcranial magnetic stimulation in refractory binge/purge behaviors. *NeuroImage Clin*. 2015;8:611–618. 26199873 10.1016/j.nicl.2015.06.008 PMC4506986

[40] Woodside DB Colton P Lam E, . Dorsomedial prefrontal cortex repetitive transcranial magnetic stimulation treatment of posttraumatic stress disorder in eating disorders: an open-label case series. *Int J Eat Disord*. 2017;50:1231–1234. 28815666 10.1002/eat.22764

[41] Pacilio RM Livingston RK Gordon MR. The use of electroconvulsive therapy in eating disorders: a systematic literature review and case report. *J ECT*. 2019;35:272–278. 31764451 10.1097/YCT.0000000000000599

[42] Andrews JT Seide M Guarda AS, . Electroconvulsive therapy in an adolescent with severe major depression and anorexia nervosa. *J Child Adolesc Psychopharmacol*. 2014;24:94–98. 24679175 10.1089/cap.2014.2422

[43] Montgomery SA Asberg M. A new depression scale designed to be sensitive to change. *Br J Psychiatry*. 1979;134:382–389. 444788 10.1192/bjp.134.4.382

[44] Busner J Targum SD. The Clinical Global Impressions Scale: applying a research tool in clinical practice. *Psychiatry (Edgmont)*. 2007;4:28. PMC2880930 20526405

[45] Baumann S Mareš T Albrecht J, . Effects of transcranial direct current stimulation treatment for anorexia nervosa. *Front Psychiatry*. 2021;12:1626. 10.3389/fpsyt.2021.717255 PMC8526853 34690831

[46] Costanzo F Menghini D Maritato A, . New treatment perspectives in adolescents with anorexia nervosa: The efficacy of non-invasive brain-directed treatment. *Front Behav Neurosci*. 2018;12:133. 30083095 10.3389/fnbeh.2018.00133 PMC6064943

[47] Khedr EM Elfetoh NA Ali AM, . Anodal transcranial direct current stimulation over the dorsolateral prefrontal cortex improves anorexia nervosa: a pilot study. *Restor Neurol Neurosci*. 2014;32:789–797. 25189181 10.3233/RNN-140392

[48] Strumila R Thiebaut S Jaussent I, . Safety and efficacy of transcranial direct current stimulation (tDCS) in the treatment of anorexia nervosa. the open-label STAR study. *Brain Stimul*. 2019;12:1325–1327. 31239105 10.1016/j.brs.2019.06.017

[49] Wideman TH Sullivan MJL Inada S, . Beck Depression Inventory (BDI). *Encycl Behav Med*. 2013;178–179.

[50] Efficacy of non-invasive vagus nerve stimulation for treatment of low weight eating disorders—full text view. ClinicalTrials.gov. Available at: https://clinicaltrials.gov/ct2/show/NCT05554172. Accessed January 7, 2023.

[51] Carroll BJ Fielding JM Blashki TG. Depression rating scales: a critical review. *Arch Gen Psychiatry*. 1973;28:361–366. 4688625 10.1001/archpsyc.1973.01750330049009

[52] Cunningham JL Wernroth L Von Knorring L, . Agreement between physicians' and patients' ratings on the Montgomery-Åsberg Depression Rating Scale. *J Affect Disord*. 2011;135:148–153. 21856017 10.1016/j.jad.2011.07.005

[53] Kearns NP Cruickshank CA McGuigan KJ, . A comparison of depression rating scales. *Br J Psychiatry*. 1982;141:45–49. 7116071 10.1192/bjp.141.1.45

[54] Möller HJ. Rating depressed patients: observer- vs self-assessment. *Eur Psychiatry*. 2000;15:160–172. 10881213 10.1016/s0924-9338(00)00229-7

[55] Dêbska E Janas A Bañczyk W, . Depression or depressiveness in patients diagnosed with anorexia nervosa and bulimia nervosa—pilot research. *Psychiatr Danub*. 2011;23(suppl 1):S87–S90. 21894110

[56] Stallwood E Monsour A Rodrigues C, . Systematic review: the measurement properties of the children's depression rating scale−revised in adolescents with major depressive disorder. *J Am Acad Child Adolesc Psychiatry*. 2021;60:119–133. 33130251 10.1016/j.jaac.2020.10.009

[57] Moneer O Daly G Skydel JJ, . Agreement of treatment effects from observational studies and randomized controlled trials evaluating hydroxychloroquine, lopinavir-ritonavir, or dexamethasone for covid-19: meta-epidemiological study. *BMJ*. 2022;377:e069400. 35537738 10.1136/bmj-2021-069400 PMC9086409

[58] Isacsson G Adler M. Randomized clinical trials underestimate the efficacy of antidepressants in less severe depression. *Acta Psychiatr Scand*. 2012;125:453–459. 22176585 10.1111/j.1600-0447.2011.01815.x

