# Supplementary material for: Anorexia Nervosa With Comorbid Severe Depression: A Systematic Scoping Review of Brain Stimulation Treatments
Source: J ECT. 2023 Apr 14;39(4):227–34. doi: 10.1097/YCT.0000000000000922 (PMC11801471; doi:10.1097/YCT.0000000000000922)
Supplement: Supplementary file 2 [file ject-39-227-s002.docx]

**Limitations of Outcome Measures Used to Measure MDD Responses**

There was a large variation in the choice of measurement instruments to assess comorbid MDD and its severity across the included treatment trials of AN patients. Items used include self-administered questionnaires and rating scales based on semi-structured interviews. The former pertain to **(1)** the Hamilton Depression Rating Scale (a score of 20 or higher is indicative of at least moderate severity MDD^1^, **(2)** the 21-item version of the Depression Anxiety Stress Scales (DASS-21)(normal (0-9p), mild (10-13p), moderate (14-20p), severe (21-27p) and extremely severe (>27p)^2^, **(3)** the Beck Depression Inventory (normal (1-10p), mild mood disturbance (11-16 p), borderline clinical depression (17-20p), moderate depression (21-30p), severe depression (31-40p) and extreme depression (>40p)^3^, **(4)** the Beck Depression Inventory II (minimal (0-13p), mild (14-19p), moderate (20-28p) and severe (29-63p)^4^. Clinician-rated measurement tools include **(1)** the Montgomery-Åsberg Depression Rating Scale (normal/symptom absent (0-6p), mild (7-19p), moderate (20-34p) or severe depression (>34p)^5^ and **(2)** the Children’s Depression Rating Scale-Revised (CDRS-R)(total score exceeding 40p is indicative of MDD, and less than 28p points to remission)^6^. The correct interpretation of any such treatment trial would be dependent on baseline MDD severity (i.e., severe MDD subjects respond differently to treatments compared to mild-moderate cases) and the accuracy of these measurements in indicating the presence of a comorbid MDD diagnosis, distinguishing MDD from other highly prevalent anxiety disorders and in assessing the longitudinal course of its severity. A study by Debska et al. underlined the complexity of diagnosing MDD in patients diagnosed with AN – positing that results of the Beck Depression Inventory needs to be confronted with the clinical picture to arrive at the correct (AN co-morbid) diagnosis^7^. None of the rating scales used have been extensively validated to measure MDD in context of clinical AN. Moreover, some of these rating scales have unsubstantiated psychometric properties to measure MDD in non-AN populations (CDRS-R)^8^ or have not adequately discriminated between MDD and anxiety disorders (DASS-21) in psychiatric patients and the general public^9^. Acknowledging the lack of designated research assessing these rating scales in their ability to adequately measure comorbid MDD in AN, it would be misguided to assume that they appropriately measure depressive symptom severity in AN patients based on preliminary evidence of differential outcomes in the aforementioned studies. Nevertheless, devoid of any comprehensively validated MDD measurement tool suitable for the target population, these rating scales may provide some heuristic value suitable for this scoping review.

**References**

1 Hamilton M. *A rating scale for depression*. J. Neurol Neurosurg Psychiatry 1960; 23:56-62

2 Lovibond S. *Manual for the depression anxiety stress scales*. 2nd ed. Psychology Foundation of Australia: Sydney N.S.W., 1995.

3 NINDS CDE Notice of Copyright Beck Depression Inventory-II (BDI-II). .

4 Beck’s Depression Inventory. http://www.med.navy.mil/sites/NMCP2/PatientServices/ (accessed 28 Aug2022).

5 Montgomery SA, Asberg M. A New Depression Scale Designed to be Sensitive to Change. *Br J Psychiatry* 1979; **134**: 382–389.

6 Mayes TL, Bernstein IH, Haley CL, Kennard BD, Emslie GJ. Psychometric properties of the Children’s Depression Rating Scale-Revised in adolescents. *J Child Adolesc Psychopharmacol* 2010; **20**: 513–516.

7 Dêbska, E, Janas A, Bañczyk W, Janas-Kozik M. Depression or depressiveness in patients diagnosed with Anorexia Nervosa and Bulimia Nervosa - pilot research. *Psychiatr Danub* 2011; **23**.

8 Stallwood E, Monsour A, Rodrigues C, Monga S, Terwee C, Offringa M *et al.* Systematic Review: The Measurement Properties of the Children’s Depression Rating Scale−Revised in Adolescents With Major Depressive Disorder. *J Am Acad Child Adolesc Psychiatry* 2021; **60**: 119–133.

9 Ali AM, Alkhamees AA, Hori H, Kim Y, Kunugi H. The Depression Anxiety Stress Scale 21: Development and Validation of the Depression Anxiety Stress Scale 8-Item in Psychiatric Patients and the General Public for Easier Mental Health Measurement in a Post COVID-19 World. *Int J Environ Res Public Health* 2021; **18**. doi:10.3390/IJERPH181910142.
