# Supplementary material for: Anorexia Nervosa With Comorbid Severe Depression: A Systematic Scoping Review of Brain Stimulation Treatments
Source: J ECT. 2023 Apr 14;39(4):227–34. doi: 10.1097/YCT.0000000000000922 (PMC11801471; doi:10.1097/YCT.0000000000000922)
Supplement: Supplementary file 3 [file ject-39-227-s003.docx]

**Supplemental table 1. Summary of brain stimulation treatment studies conducted in the context of anorexia nervosa.**

|  |  |  |  |  |  |  |  | **BMI (kg/m^2^)** | | | |  | **MDD** | | | | |
| --- | --- | --- | --- | --- | --- | --- | --- | --- | --- | --- | --- | --- | --- | --- | --- | --- | --- |
|  |  |  |  |  |  |  |  |  |  |  |  |  |  |  |  |  |  |
|  |  |  |  |  |  |  |  |  |  |  |  |  |  | **Baseline*** | |  | **Follow-up*** |
| **Treatment modality** | **Author and Year** | **Type of study** | **n, ED diagnosis** | **Inpatient/Outpatient** | **Age (years), Gender*** | **Psychiatric Comorbidity** | **Treatment duration** | **BMI outcome reported (Y/N)** | **Baseline*** | **AN-severity (inferred from baseline BMI)** | **Follow-up (absolute change in BMI/weight from baseline, (BMI/weight at follow-up))*** |  | **MDD outcome reported  (Y/N, instrument)** | **Score** | **Inferred MDD severity** |  | **Score (absolute change from baseline (score))** |
|  |  |  |  |  |  |  |  |  |  |  |  |  |  |  |  |  |  |
| rTMS | Kamolz et al. , 2008^1^ | Case report | 1, AN | Outpatients | 24, female | Severe MDD | 20 weeks / 41 sessions | Y | >13 | Extreme | > 3 (**BMI**: 16) |  | Y, HMDRS | 28 | Moderate-Severe MDD |  | PT: -18 (10=remission) |
| rTMS | Van den Eynde, et al. , 2013^2^ | Case series (pilot study) | 7, AN-R 2, AN-B/P | Mixed, mainly outpatients | Median: 25 (range 18-44). Gender not explicitly reported. | Not reported | 1 session | N | **Mean**: 15.7 (range: 13.8-17.8) | Extreme-Severe-Moderate-Mild | Not reported |  | N, DASS-21 (total score) | *depression subscale not reported | - |  | - |
| rTMS | McClelland et al., 2013^3^ | Case report | 1, AN-R 1, AN-B/P | Not explicitly stated, at least 1 outpatient | 23 and 52, females | 23 yr-old: **MDD** (severity not specified). | 19-20 sessions *total duration not explicitly stated | Y | 15.76 and 16.40 | Severe and Moderate | **Post-treatment (PT)**: -0.3, 0.04 (BMI: 15.46, 16.44)  **1-month PT**: -1.02, -0.41 (BMI: 14.74, 15.99) |  | Y, DASS-21 | **Patient 1**: 4 (depression subscale) **Patient 2**: 10 (depression subscale) | **Patient 1**: Normal/No MDD **Patient 2**: Mild MDD |  | **Patient 1**: -2 (2) [**PT**], 2 (6) [**1 mo PT**] **Patient 2**: 1 (11) [**PT**], -3 (7) [**1 mo PT**] |
| rTMS | McClelland et al., 2016^4^ | Case series | 2, AN-R/P 2, AN-B/P 1, AN-R | Not explicitly stated | 23, 30, 41 and 52; females | Not reported | 20 sessions *total duration not explicitly stated | Y | 14.78, 16.40, 19.24, 15.35, 14.54 | Extreme, moderate, mild, severe, extreme | **PT**: -0.28 (BMI: 14.5), 0.05 (BMI: 16.45), 0.11 (BMI: 19.35), 0 (BMI: 15.35), N/A (BMI: not reported) **6 mo post PT**: -0.96 (BMI: 13.82), -0.62 (BMI: 15.78), -1.36 (BMI: 17.88), -1.39 (BMI: 13.96), -0.65 (BMI: 13.89) **12 mo post PT**: -0.92 (BMI: 13.86), -1.14 (BMI: 15.26), -1.21 (BMI: 18.03), -1.39 (BMI: 13.96), -0.46 (BMI: 14.08) |  | N, DASS-21 (total score) *depression subscale not reported | *depression subscale not reported | - |  | *depression subscale not reported |
| rTMS | McClelland et al., 2016^5^ | RCT | **Treatment group (n=21)**: 13 AN-R 8 AN-B/P  **Sham-control group (n=28)**: 15, AN-R 13, AN-B/P | Not explicitly stated, implicit outpatients | **rTMS-group**: 25.29 yr (SD=6.88)  **Sham-group**: 27.68 (SD=9.89) **Gender**: Females | Not reported | 1 session | N | **rTMS-group (Mean (SD))**: 16.73 (1.59) **Sham-group (Mean (SD))**: 16.38 (1.76) | Moderate *including also outlier samples with mild and severe inferred severity | Not reported |  | N, DASS-21 (at baseline only) | **rTMS group**: 10.14 (SD=5.98)  **Sham-control group**: 12.00 (SD=5.92)  *Mean of depression subscale | **rTMS-group**: Mild MDD **Sham-control group**: Mild MDD *rTMS-group including also outlier samples with moderate and normal/no inferred MDD severity *Sham-group including also outlier samples with moderate and normal/no inferred MDD severity |  | Not reported |
| rTMS | Choudhary et al., 2017^6^ | Case report | 1, AN | Inpatients | 23, female | Not reported | 21 sessions *total duration not explicitly stated | Y | 14.74 | Extreme | **PT**: 3.24 (BMI: 17.98) **8 wk PT**: 3.81 (BMI: 18.55) |  | N | Not reported | - |  | - |
| rTMS | Jassova, 2018^7^ | Case report | 1, AN | Inpatients | 25, female | **MDD** (severity not specified), **anxiety** | 10 sessions *total duration not explicitly stated | Y | 11.98 | Extreme | **PT**: 0.15 (BMI: 12.13) **At discharge**: 1.17 (13.15) |  | Y, Clinical assessment | Not reported | - |  | No improvement of depression |
| rTMS | Woodside, 2017^8^ | Case series | 2, AN-R 4, AN-B/P | Mixed | Mean: 39.8 (SD=10.9), females *full sample including ENDOS | **PTSD**: Full sample **MDD**: 6 **Bipolar Disorder**: 2 **Social Anxiety**: 3 **Borderline Personality Disorder**: 1 **Panic disorder**: 1 **Obsessive-compulsive disorder**: 1 *full sample including ENDOS | 20-30 sessions *total duration not explicitly stated | N | 20.81 *full sample including EDNOS | Mild | Not reported |  | N, - | Not reported | - |  | Not reported |
| rTMS | Dalton et al., 2018^9^ | RCT | **Treatment group (n=17)**:  11, AN-R 6, AN-B/P  **Sham-control group (n=17)**: 11, AN-R 6, AN-B/P | Mixed, mainly outpatients | **rTMS-group**: 28.47 (SD=9.48) **Sham-group**: 31 (SD=11.29) **Gender**: Females | Not reported | 20 sessioner *total duration not explicitly stated | Y | **rTMS-group (Mean (SD))**: 15.76 (1.62)  **Sham-group (Mean (SD))**: 16.26 (1.22) | **rTMS-group**: Severe **Sham-group**: Moderate *rTMS-group including also outlier samples with moderate and extreme inferred severity *Sham-group including also outlier samples with moderate and severe inferred severity | **PT**: 0.11 (BMI: not explicitly reported), 0.08 (BMI: not explicitly reported) **3 mo post PT**: 0.28 (BMI: not explicitly reported), 0.04 (BMI: not explicitly reported) |  | Y, DASS-21 | **rTMS group:** 26.82 (SD=9.44) (Mean of depression subscale) **Sham-control group:** 25.38 (SD=10.19) (Mean of depression subscale) | **rTMS group:** severe MDD  **Sham-control group:** severe MDD *rTMS-group including also outlier samples with moderate and extreme inferred MDD severity *Sham-group including also outlier samples with moderate and severe inferred MDD severity |  | **PT_Mean Change rTMS group_**: -5.13 (SD=8.94) **PT_Mean Change Sham-control group_**: -3.25 (SD=10.55) **3 mo PT_Mean Change rTMS group_**: -9.31 (SD=10.61)  **3 mo PT_MeanChange Sham-Control group_**: -1.13 (SD=8.58) |
| rTMS | Khnyakhnytska et al., 2019^10^ | Case series (pilot study) | 6, AN-R 2, AN-B/P | Outpatients | Mean: 33 (SD=11.56), females | Not reported | 42 sessioner *total duration not explicitly stated | Y | **Mean**: 16.6 (SD=0.9) | Moderate *including also outlier samples with mild and severe inferred severity | **6 wk PT**: 0.46 (BMI: 17.06) **12 wk PT**: -0.27 (BMI: 16.33) **6 mo PT**: 0.38 (BMI: 16.98) |  | Y, HDRS, MADRS (clinician-assessed), BDI | **HDRS (Mean)**: 12.22 (SD=5.05) **MADRS (Mean)**: 17.88 (SD=5.25) **BDI (Mean)**: 24.66 (SD=9.97) | **HDRS**: Normal/No MDD  **MADRS**: Mild MDD **BDI**: Moderate MDD |  | **PT:** HDRS: -5.39 (6.83 (SD=5.36)), MADRS: -6.28 (11.6 (SD=8.93)), BDI: -11.86 (12.8 (SD=12.7)) **6 wk PT:** HDRS: -6.72 (5.5 (SD=4.7)), MADRS: -6.88 (11 (SD=7.9)), BDI: -14.66 (10 (SD=11.3)) **4.5 mo PT**: HDRS: -1.97 (10.25 (SD=5.1)), MADRS: -2.88 (15 (SD=5)), BDI=-11.41 (13.25 (SD=13.3)) |
| rTMS | Dalton et al., 2020^11^ | RCT | **Treatment group (n=13):** 9, AN-R 4, AN-B/P  **Sham-control group (n=13):** 9, AN-R 4, AN-B/P | Mainly outpatients | **rTMS-group**: Mean: 30.20 (SD=10.00) **Sham-group**: 31.30 (SD=12.50) **Gender**: Females | Not reported | 20 sessions *total duration not explicitly stated | Y | **rTMS-group (Mean (SD))**: 15.70 (1.40) **Sham-group (Mean (SD))**: 16.30 (1.20) | **rTMS-group**: Severe **Sham-group**: Moderate *rTMS-group including also outlier samples with moderate and extreme inferred severity *Sham-group including also outlier samples with moderate and severe inferred severity | **PT**: -0.1 (BMI: 15.60), -0.1 (BMI: 16.20) **4 mo PT**: 0 (BMI: 15.70), -0.2 (BMI: 16.10) |  | Y, DASS-21 (only depression subscale reported) | **rTMS group**: 27.10 (SD=9.50) **Sham-control group**: 24.60 (SD=9.98) | **rTMS-group**: severe MDD **Sham-control group**: severe MDD *rTMS-group including also outlier samples with moderate and extreme inferred MDD severity *Sham-group including also outlier samples with moderate and severe inferred MDD severity |  | **PT_rTMS group_**: -5.1 (22.00 (SD=10.80)) **PT_Sham-control group_**: -3.1 (21.50 (SD=9.10)) **4 mo PT_rTMS group_**: -9.9 (17.20 (SD=10.20)) **4 mo PT_Sham-control group_**: -1.7 (22.90 (SD=13.20)) |
| rTMS | Muratore, 2021^12^ | Cross-over trial (sham/rTMS) | 10 AN (7 AN-R) | Inpatients | 30.7 (SD=7.4), females only | Not reported | 1 session 10 pulses/s 4-trains 120% MT, 3000 pulses) (high-frequency) DLPFC | N | 17.1 (SD=1.8) | Mild | N/A |  | N | - | - |  | - |
| rTMS | Dunlop et al., 2015^13^ | Case series | 11, AN-BP | Not explicitly stated | Mean: 31.9 (SD=9.5), range 20-56 Gender: Females *full sample including 17 BN patients | **MDD**: 16 **PTSD**: 8 **Obsessive-compulsive disorder**: 6 **Bipolar Disorder**: 6 *full sample including 17 BN patients | 20-30 sessions *total duration not explicitly stated | N | Mean: 19.03 (SD=5.33) *full sample including 17 BN patients | Mild *Including also outlier samples with extreme, severe and/or moderate severity | Not reported |  | N, HDRS & BDI-II (scores not reported) | - | - |  | - |
| ECT | Bernstein, 1964^1^ | Case report | 1, AN | Inpatient | 19, female | **MDD** (severity not specified) "**Schizophrenic reaction" ***with "paranoid and affective features" | 21 sessions *electrode placement, frequency and total duration not explicitly stated | Y | 75 lbs *length/BMI not reported | - | **Post 10 sessions**: 39 lbs (BMI: not reported)* **Post 21 sessions**: 16 lbs (BMI: not reported)* |  | N | - | - |  | Not reported |
| ECT | Ferguson et al, 1993^2^ | Case report | 3, AN | Inpatients | 23, 35 & 42, females | **Patient 1**: non-MDD **Patient 2**: MDD (severity not specified) *reported 'neurovegetative signs' and prior suicide attempt **Patient 3**: MDD (severity not specified) *reported recent inpatient suicide attempt | 8, 11 & 16 bilateral sessions *frequency and total duration not explicitly stated | Y | 60 lbs, 64 lbs, 92 lbs *length/BMI not reported | - | **PT**: 17 lbs, 20 lbs, 8 lbs  **Follow up:** 30lbs (3-yr PT), 31 lbs, (1-yr PT), 23 lbs (2-yr PT)  *Follow up measurements of BMI reported at different timepoints for each case. *length/BMI not reported |  | N | - | - |  | Not reported |
| ECT | Hill et al, 2001^3^ | Case report | 1, AN-R | Inpatient | 77, female | **MDD** | 9 sessions *electrode placement, frequency and total duration not explicitly stated | Y | 40 kg *length/BMI not reported | - | **PT**: 2 kg (BMI: Not reported)* |  | Y , Clinical Assessment | - | **MDD** (severity not specified) |  | Subjective mood improvement |
| ECT | Poutanen et al., 2009^4^ | Case report | 1, AN-B/P | Inpatient | 21, female | **MDD** | 10 + 12 bilateral sessions and 23 bilateral maintenance ECT sessions *frequency and total duration not explicitly stated | Y | **Initial treatment series (baseline)**: 15.0 **Second treatment series (baseline #2)**: 14.2 **Maintenance (baseline #3):** 14.6 | Severe | **PT (initial series)**: 0.3 (BMI: 15.3) **PT (latter series)**: -0.4 (BMI: 14.6) **PT (Maintenance)**: +1.8 (BMI: 16.4) |  | Y, BDI and MADRS (only at second series) | **BDI_First series_**: 52 **BDI_Second series_**: 35 **MADRS_First series_**: Not reported **MADRS_Second series_**: 28 | Extremely severe MDD |  | **PT_First series_ (BDI):** -17 (35) **PT_Second series_ (BDI):** -9 (43) **PT_First series_ (MADRS)**: Not reported **PT_Second series_ (MADRS)**: -4 (24) |
| ECT | Andrews et al., 2014^5^ | Case report | 1, AN-B/P | Inpatient | 17, female | **MDD, NSSI** | 31 sessions over 13 wks *10 right unilateral followed by 21 bilateral ECT sessions *frequency not explicitly stated | N | Pre-treatment  *BMI not reported | - | Weight stabilization |  | N, Clinical Assessment | **-** | **-** |  | Subjective reduction in depressive symptoms and self-harm |
| ECT | Andersen et al., , 2017^6^ | Case report | 1, AN-R | Mixed | 14, female | **MDD, GAD** | 22 bilateral sessions (13 during hospitalization episode) *electrode placement, frequency and total duration not explicitly stated | Y | 50.1 kg *length/BMI not reported. Weight reported at admission, not at treatment initiation | - | **At discharge**: 9.7 kg (BMI: not reported at baseline) **Follow-up**: 13.6 kg (BMI: not reported at baseline) |  | Y, CDRS-R | 43 | **MDD** (severity not specified) |  | **PT**: -26 (17 = remission) |
| ECT | Saglam, 2018^7^ | Case report | 1, AN-B/P | Not explicitly stated | 24, male | **OCD, MDD** | 12 sessions *electrode placement, frequency and total duration not explicitly stated | Y | 16.5 | Mild-Moderate | **PT**: 4 (BMI: 20.5) |  | Y, HMDRS | 23 | Not indicative of at least moderate MDD |  | -13 (10) |
| ECT | Pacillo et al., 2019^8^ | Case report | 1, AN | Inpatient | 30, female | **MDD** | 11 sessions *electrode placement, frequency and total duration not explicitly stated | Y | 15.2 | Severe | **PT**: 0.5 (BMI: 15.7) |  | Y, MADRS | 33 | Borderline severe MDD |  | **PT**: -23 (10=remission) |
| ECT | Naguy et al, 2019^9^ | Case report | 1, AN | Inpatient | 16, female | **Unspecified Personality Disorder** | 6 sessions *electrode placement, frequency and total duration not explicitly stated | Y | 16 | Severe | **PT**: 4 kg weight increase (BMI: not reported at follow-up) |  | N | **-** | **-** |  | - |
| ECT | Duriez et al, 2020^10^ | Case report | 1, AN | Inpatient | 21, female* | None | 10 sessions *Twice-weekly brief-pulse bilateral electrode placement (pulse widts, 0.50 milliseconds; frequency 30 Hz, duration 6.5 seconds, current 0.91A) for a total duration of 5 weeks | N | Not explicitly stated | - | **2 mo PT**: N/A (BMI 19.6) *BMI not reported at baseline |  | Y, HADS-depression | 10 | Indicative of low mood, not clinical MDD requiring medical treatment |  | **PT**: -1 (9) |
| ECT | Shilton et al, 2020^11^ | Case series | 14, AN-R 16, AN-B/P | Inpatients | Mean 16.1 (SD)=1.6 **Gender**: Females | **Severe MDD**: Full sample **OCD**: 11 **Anxiety**: 7 **PTSD**: 6 **NSSI**: 28 | Mean number of sessions: 16.7 (SD=6.8) *At initiation, twice-weekly treatments with bitemporal electrode placement (gradually reduced to once-weekly and then once-monthly sessions) *total duration not explicitly stated | Y | **Mean_On Admission_**: 17.34 (SD=1.93, range=12.8-22.3) | Mild | **PT**: 2.8 (BMI_Mean_: 20.14 (SD=1.22)) |  | Y, CGI-S (Clinical Global Impression-severity scale) | 5.95 (SD=0.5) | Severe MDD |  | **PT:** -2.07 (3.88 (SD=0.9)) **At discharge: -**2.27 (3.68 (SD=1.04)) |
| ECT | Davis 1961^12^ | Case | 1 AN-R | Inpatient* | 12 yr, female | **MDD** (severity not specified, reported 'suicidal ideation') | 12 bilateral sessions *Frequency and total duration not reported | Y | 58 lbs | - | 12.5 lbs (70.5 lbs) |  | N | - | - |  | - |
| ECT | Bernstein 1972^13^ | Case | 1 AN-R | - | 94 yr, female | Schizophrenia | 5 sessions  *electrode placement, frequency and total duration not reported | Y | - | - | 5 lbs weight gain in 9 days |  | N, Clinical Assessment | - | - |  | Subjective mood improvement |
| ECT | Bek & Hotujak 1996^14^ | Case series | 8 AN, 2 treated with ECT | Unknown | 16-22 yr, females | Unspecified personality disorder: 4 Paranoid psychosis: 1 *including full sample, of which 2 received ECT | Not reported |  | 45.9 kg mean *including full sample, of which 2 received ECT | - | All but one patient gained weight, 6 <10% gain, 1 >10%. |  | N | - | - |  | - |
| tDCS | Khedr et al., 2014^1^ | Open-label, single arm study (pilot study) | 7, AN | Not explicitly stated | Mean 21.75 (SD=7.8, range 16-39),  **Gender**: 6 females & 1 male | **Severe MDD**: 3 **Mild MDD**: 2 **Anxiety**: 3 *some patients exhibited multiple comorbidities | 10 sessions *total duration not explicitly stated | N | **Patient 1**: 15 **Patient 2**: 15 **Patient 3**: 12 **Patient 4**: 17 **Patient5**: 14  **Patient 6**: 16 **Patient 7**: 15 *Range: 12 - 17 | 2 Extreme 3 Severe  1 Moderate  1 Moderate/Mild | 5/7 patients exhibiting improvements on eating attitude test, 4/7 maintaining response at 1 month *BMI not reported at follow-up |  | Y, BDI-II | 39, 19, 36, 29, 8, 10, 16 | 2 severe MDD 1 moderate MDD 2 mild MDD 2 minimal/no MDD |  | 6/7 patients considered improved on the BDI at PT (3/7 at follow-up) *PT and follow-up scores not reported, illustrated in graph |
| tDCS | Strumila, 2019^2^ | Open-label study | 10, AN | Inpatients | Age not reported, females | Not reported *subjects received medication for putative comorbid conditions | 2 weeks, 20 sessions | N | Not reported | - | Anorexic symptoms reportedly improved *BMI not reported at follow-up |  | Y, BDI | BDI: 16.89 (SD=5.16), C-SSRS 6.67 (SD=8.53) | Borderline clinical depression |  | PT (BDI): -4.22 (12.67 (SD=5.20)) PT (C-SSRS): Not reported 1 mo PT (BDI): -2.89 (14.00 (SD=6.96)) 1 mo PT (C-SSRS): 0.77 (7.44 (SD=9.85)) |
| tDCS | Costanzo, 2018^3^ | RCT | 23 AN (11 active tDCS-group [1 male], 12 FBT-control group [0 males] | Not explicitly stated, putatively outpatients | tDCS-group: 13.9 (SD=1.8), Control: 15.1 (SD=1.5). 22 females, 1 male | Not stated | 1mA tDCS (20 min), 3 times weekly for 6 weeks (Total: 18 sessions). Anodal electrode left DLPFC, cathodal right DLPFC | Y | tDCS: 14.7 (SD=2.2) Control: 15.5 (SD=1.6) | Active: Extreme, Control: Severe | tDCS: 1.9 (16.6, SD=2.3)  Control: 0.6 (16.1, SD=1.3).  Significant difference. Taken post-measurement. Increase in BMI persisted in 9 tDCS-participants measured 1-month later |  | CDI, Children's depression inventory | tDCS: 23.2 (SD=15.5) Control: 20.8 (SD=7.9) | - |  | tDCS: -11.8 (11.4, SD=9.5) Control: -7.6 (13.2, SD=6.4).  Difference not significant. |
| tDCS | Bauman, 2021^4^ | RCT (Sham-control) | 39 AN 4 atypical AN (33 included in statistical analysis, 17 active, 16 sham-control) | Inpatient | tDCS group=23.7 (SD=6.38), Sham= 28.1 (SD=7.95). Females only | tDCS group: 3 MDD, 3 Anxiety, 2 personality disorder Sham-control: 4 MDD, 6 Anxiety, 5 history of substance abuse, 3 personality disorder | Ten 30-min session 2mA anodal stimulation, left DLPFC | Y, (absolute BMI values not reported at follow-up) | tDCS: 16 (sd=1.69), Control 16.8 (sd=2.47) | Moderate | Described as improved but not significant in active and control groups at measurements at end of treatment of 2-week follow-up 4-week follow-up |  | Y, Zung-Self-Rating Depression Scale | Active group: 71.6 (SD=8.57)  Control: 72.3 (SD=11.4) | Severe MDD |  | The sham-group exhibited greater reductions in the self-rated depression scale (values not reported). |
| DBS | Israel et al., 2010^1^ | Case report | 1, AN-R | Not explicitly stated | 56, female | **Severe MDD** | Approx. 2 1/2 years | Y | 20.9 | Mild | **PT**: -1.8 (BMI: 19.1) |  | N | - | - |  | - |
| DBS | Barbier et al., 2011^2^ | Case report | 1, AN | Inpatients | 38, female | **OCD** | 1 year | Y | 13.1 | Extreme | **2 wk post-op (PO)**: 0.6 (BMI: 13.7) **3 mo PO**: 9.9 (BMI: 23) **1 yr PO**: 9.2 (BMI: 22.3) |  | Y, MADRS | MADRS: 20 | moderate MDD |  | **3 mo PO**: -10 (10) |
| DBS | Mclaughlin et al. , 2013^3^ | Case report | 1, AN | Not explicitly stated | 52, female | **OCD** | Not stated | Y | 18.5 | Mild | +0.4-1.1 (Stated as a range post-op) *exact duration PO of measurements not specified |  | N | - | - |  | - |
| DBS | Wu et al. , 2013^4^ | Case series | 4, AN | Not explicitly stated | 16.5 (range: 16-17), females | **OCD**: 3 **GAD**: 1 *No individual with multiple reported comorbidities | Average follow up 38 months (range 9-50) | Y | 12.2, 13.3, 12, 10 | Extreme | **PT**: 7.73 (BMI_Mean_: 19.6) *Only mean BMI reported at follow-up |  | N | - | - |  | - |
| DBS | Wang et al., 2013^5^ | Case series | 8, AN | Inpatients* | 18 & 27, females | Not reported | 1 year | Y | **Patient 1**: 13.3 **Patient 2**: 12.9 | Extreme | **1 wk PO**: 0.2 and 0.3 (BMI: 13.5 and 13.2) **6 mo PO**: 3.8 and 6.3 (BMI: 17.1 and 19.2) **1 yr PO**: 4.7 and 7.9 (BMI: 18 and 20.8) |  | Y, HAM-D | 24, 20 | At least moderate severity MDD |  | **1 wk PO: -5 (19), -4 (16) 6 mo PO: -**11 (13), -12 (8) **1 yr po**: -15 (9), -11 (9) |
| DBS | Zhang et al., 2013^6^ | Case series (neuroimaging) | 4 AN-R | Not specified | Mean age=17 (SD=0.8)(included patients who did not go through DBS), females only | Not specified | Nucleus accumbens | Y | 11.8, 11.2, 13.3, 12.2 | Extreme | **1-month post-OP**: 6.1 (17.9), 1.9 (13.2), 1.2 (14.5), 4.9 (17.1) |  | N | - | - |  | - |
| DBS | Lipsman et al., 2013^7^ | Open label clinical trial | 1, AN-P 1, AN-B/P 4, AN-R | Not explicitly stated | Mean: 38 (SD=11) **Gender**: Females | **MDD (severity not reported)**: 4 **OCD**: 4 **PTSD**: 2 **SUD**: 1 | 9-month | Y | 16.1 (SD=1.5) | Moderate *including also outlier samples with mild and severe inferred severity | **2 mo PO**: -2.1 (BMI_Mean_: 14.0) **4 mo PO**: -1.4 (BMI_Mean_: 14.7) **6 mo PO**: -0.8 (BMI_Mean_: 15.3) **9 mo PO**: 0.5 (BMI_Mean_: 16.6) |  | Y, HAM-D & BDI | **HAMD (Pre-op)**: 17.8 (SD=8.2)  **BDI (Pre-op)**: 38.8 (SD=23.2) | **HAMD**: No MDD - mild MDD **BDI**: Severe MDD *HAMD: including also outlier samples with mild and severe inferred MDD severity *BDI: including also outlier samples with borderline clinical depression and extreme inferred MDD severity |  | **1 mo PO**: HAMD: -4.2 (13.6 (SD=6.6)) BDI: -9.6 (29.2 (SD=6.2)) **3 mo PO**: HAMD: -5.3 (12.5 (SD=6.6)) BDI: -13 (25.8 (SD=18.3)) **6 mo PO**: HAMD: -7.1 (10.7 (SD=8.4) BDI: -18.6 (20.2 (SD=20.-4)) |
| DBS | Hayes et al. , 2015^8^ | Ancillary Study | 4, AN 3, AN-B/P 1, AN-R | Not explicitly stated | Mean: 35 (SD=11).  **Gender**: Females | **MDD**: 7 **PTSD**: 5 **OCD**: 2 **GAD**: 1 **Unspecified Anxiety Disorder**: 1 | 1 year | Y | Not explicitly stated | - | **12 mo post baseline (pre-op)**: Mean of % change 22.5% (SD: 20.1%) **12 mo PO**: Mean of % change 7% (SD: 21.4%) *Absolute BMI values not reported on an individual basis post-treatment |  | Y, HAM-D & BDI | Not reported | - |  | **12 mo PO**: HAMD: -35 %, -76 %, -67%, +23, -86, -50, -80, -70 BDI: -21 %, -29 %, -28%, -16%, -100%, -50%, -82, +39 *6 patients reported with clinically improved MDD, 1 deteriorated and 1 unchanged (across both HAMD and BDI, albeit two different individuals deteriorated on the HAMD and BDI, respectively) |
| DBS | Lipsman et al., 2017^9^ | Open label clinical trial | 9, AN-R 6, AN-BP 1, AN-P | Mixed | Mean: 34 (SD=8) **Gender**: Females | **MDD (severity not reported)**: 12 **PTSD**: 10 **OCD**: 5 **GAD**: 3 **Unspecified Anxiety Disorder**: 2 | 1 year | Y | 13.83 (SD=1.49) | Extreme *including outlier samples with severe inferred severity | **1 yr PO**: 3.51 (BMI: 17.34) |  | Y, HAM-D, BDI | **HAMD**: 19.40 (SD=6.76) **BDI**: 42.94 (SD=15.76) | **HAMD**: Mild to moderate MDD **BDI**: extreme MDD severity *BDI: Including also samples with moderate and severe inferred MDD severity |  | **1 mo PO**: HAMD: -5.4 (14.0 (SD=6.7)) BDI: -7.44 (35.5 (SD=14.8) **3 mo PO**: HAMD: -7.76 (11.64 (SD=7.8)) BDI: -15.14 (27.8 (SD=17.4)) **6 mo PO**: HAMD: -6.7 (12.7 (SD=9.7)) BDI: -19.24 (23.7 (SD=18.9)) **12 mo PO**: HAMD: -10.60 (8.8 (SD=7.6)) BDI: -17.8 (25.14 (SD=18.32)) |
| DBS | Blomstedt et al., 2017^10^ | Case report | 1, AN | Inpatients | 60, female | **MDD (severity not reported)** *Exhibited "*significant symptoms of anxiety"*, no report of any anxiety disorder diagnosis | 36-months | Y | **Initial surgery (baseline)**: 16.2  **Second surgery (baseline #2)**: 15.2 | Moderate | **6 mo PO_First op_**: 0.0 (BMI: 16.2) **12 mo PO_First op_**: 0.3 (BMI: 16.5) **24 mo PO_First op_**: -1.0 (BMI: 15.2) **6 mo PO_Second op_**: -1.7 (BMI: 14.5) **12 mo PO_Second op_**: -1.9 (BMI: 14.3) |  | Y, HAM-D, MADRS | **MADRS_First Op_**: 43 **MADRS_Second Op_**: 27 **HAM-D_First Op_:** 22 **HAM-D_Second Op_**: 15 | **MADRS**: severe MDD **HAM-D**: At least moderate severity MDD |  | **6 mo PO_First op_:** MADRS: -17 (26) HAM-D: 0 (22) **12 mo PO_First op_:** MADRS: -10 (33) HAM-D: 8 (30) **24 mo PO_First op_:** MADRS: -16 (27) HAM-D: -7 (15) **6 mo PO_Second op_:** MADRS: -29 (14) HAM-D: -7 (15) **12 mo PO_Second op_:** MADRS: -30 (13) HAM-D: -16 (6) |
| DBS | Manuelli et al., 2020^11'^ | Case report | 1, AN | Mixed | 37, female | **OCD** | 4-months | Y | 16.31 | Moderate | **4 mo PO**: 2.16 (BMI: 18.47) |  | N | - | - |  | - |
| DBS | Wei Liu et al., 2020^12^ | Open label clinical trial | 13, AN-R 15, AN-BP | Not explicitly stated | Mean: 22.8 (SD=4.1) **Gender**: Female | **MDD (severity not reported)**: 12 **OCD**: 9 *7 subjects with "severe anxiety", no explicit report of any anxiety disorder diagnosis | 12-months | Y | 13.01 (SD=1.86) | Extreme | **6 mo PO**: 2.28 (BMI_Mean_: 15.29) **12 mo PO**: 4.72 (BMI_Mean_: 17.73) |  | Y, HAM-D | 26.93 (SD=11.97) | At least moderate severity MDD |  | **6 mo PO**: -10 (16.93 (SD=10.93)) **2 yr PO**: -11 (15.93 (SD=12.33)) |
| DBS | Fernandes Arroteia, et al. 2020^13^ | Case study | AN-BP (Beskrivs som bulimic type) | Not specified | 42, female | MDD | Nucleus accumbens | N | 12.8 (32 kg) | Extreme | 15 kg increase (47 kg) from baseline during 4 measurements: 12, 14, 15 and 18 months post op. 10 kg increase from baseline (42 kg) at measurement 24 months post op. |  | Y, clinical assessment | comorbid MDD | Not reported |  | Assessed as no improvement. |
| DBS | Lin, 2020^14^ | Case in ancillary study | 1 AN (SE-AN) | Outpatient | Not stated | Not specified | Bed nucleus of the stria terminalis | N | - | - | - |  | Y, HAMD | 27 | At least moderate |  | ´-2 (25) at 2 months. |
| DBS | De Vloo et al, 2021^15^ | Case series | 9, AN-R, 5 AN-BP, 1 AN-P | Not specified | 34 (SD=9), females only | **MDD (severity not reported)**: 12  **PTSD**: 9 **OCD**: 5 **GAD**: 2 **Anxiety NOS**:2 **BPD**: 1 **SUD**: 1 | Subcallosal cingulate | Y | 14.0 | Extreme | 1-year: +3.5 (17.5) 3-year: 2.3 (16.3) |  | Y HAMD, BDI | Not reported | N/A |  | Significant change in both HAMD and BDI at 1-year follow-up, although not maintained at 3-years. 44 % met criteria for clinical response on HAMD at 3-years. |
| DBS | Oudijn et al., 2021^16^ | Case series | 4, AN not specified | Not specified | 39 (SD=10), females only | Not specified | Ventral anterior limb of the capsula interna | Y | 12.70 (SD=1.71) | Extreme | 5.06 (mean: 17.76, SD=1.71) measured 15-21 months post-op |  | Y HAMD | 27.25 (SD=2.09) | At least moderate |  | 15-21 mo PO: -10 (mean: 17.25 , SD=2.09) |
| DBS | Scaife, 2022^17^ | Pilot study (Two consecutive, randomized blind on-off fortnights 9 months after stimulation onset) | 7 restrictive AN | 1 inpatient, not specified | 37 (SD=13.7), 6 females and 1 male | **MDD (severity not specified)**: 5 **OCD**: 4 **GAD**: 1 **OCPD (OC-personality disorder)**: 1 | Nucleus accumbens | Y | 15.2 (SD=1.5) | Severe | 3 mo PO: 0 (mean: 15.2, SD=0.9) 6 mo PO: -0.2 (mean: 15.0, SD=1.3)  12 mo PO: 0.1 (mean: 15.3, SD=1.5) |  | Y, HAMD | 13.2 (SD=5.6) | Not indicative of MDD |  | 3 mo PO: -0.3 (mean: 12.9, SD=11.2) 6 mo PO: 3.4 (mean: 16.6, SD=10.4) 12 mo PO: -0.6 (mean: 12.6, SD=9.0). Difference not significant |
| DBS | Martinez et al., 2020^18^ | Open label clinical trial | 6, AN-R 1, AN-B/P 1, AN-P | Mixed | Mean: 40.75 (SD=15.49) **Gender**: 7 females, 1 male | **MDD (severity not reported)**: 7  **OCD**: 3 **Panic Disorder**: 3 | 6-months | Y | 12.67 (SD=1.64) | Extreme | **6 mo PO**: 1.31 (BMI_Mean_: 13.98) |  | Y, HAM-D | 15.38 (SD=5.52) | Not indicative of MDD |  | **6 mo PO**: -4.88 (10.50 (SD=9.87)) |

**Supplemental table 1. Legend**: This table summarizes the literature reviewed in this review regarding the efficacy of repetitive transcranial magnetic stimulation (rTMS), direct transcranial current stimulation (tDCS), electroconvulsive therapy (ECT) and direct brain stimulation (DBS), with a focus on outcomes in body weight and depressive symptomatology. Anorexia severity was inferred based on reported BMI. Severity of depression was inferred based on cut-off rates of reported scores on psychometric instruments. In the absence of this, severity estimations based on clinical estimations were accepted. In articles with multiple measurement points, outcomes were contrasted with the first reported baseline value.

*Abbreviations*: AN: anorexia nervosa, AN-B/P: anorexia nervosa binge/purging subtype, AN-R: anorexia nervosa restricting subtype, AN-P: Anorexia nervosa purging subtype, BDI: Beck Depression Inventory; CGI-S : Clinical Global Impression-Severity Scale: DASS-21: Depression Anxiety and Stress Scale: DLPFC: Dorsolateral prefrontal cortex, DMPFC: Dorsomedial prefrontal cortex, HAMD or HDRS: Hamilton Depression Rating Scale, EDNOS: eating-disorder not otherwise specified, GAD: generalized anxiety-disorder, MADRS: Montgomery-Åsberg Depression Rating Scale, MDD: major depressive disorder, mo: months, OCD: obsessive-compulsive disorder, PT: post-treatment, PTSD: post-traumatic stress disorder, PO: post-operation.

**References (Supplementary Table 1 - rTMS)**

1. Kamolz, S., Richter, M., Schmidtke, A. *et al.* Transkranielle Magnetstimulation gegen komorbide Depression bei Anorexie. *Nervenarzt* **79,**1071 (2008). https://doi.org/10.1007/s00115-008-2537-8
2. Van den Eynde, F., Guillaume, S., Broadbent, H., Campbell, I. ., and Schmidt, U. (2011). Repetitive transcranial magnetic stimulation in anorexia nervosa: A pilot study. *European Psychiatry*, *28*(2), 98–101. <https://doi.org/10.1016/j.eurpsy.2011.06.002>
3. McClelland, J., Bozhilova, N., Nestler, S., Campbell, I. C., Jacob, S., Johnson-Sabine, E., and Schmidt, U. (2013). Improvements in Symptoms Following Neuronavigated Repetitive Transcranial Magnetic Stimulation (rTMS) in Severe and Enduring Anorexia Nervosa: Findings from two Case Studies: Improvements in symptoms of severe and enduring anorexia with rTMS. *European Eating Disorders Review*, *21*(6), 500–506. <https://doi.org/10.1002/erv.2266>
4. McClelland, J., Kekic, M., Campbell, I. C., and Schmidt, U. (2016). Repetitive Transcranial Magnetic Stimulation (rTMS) Treatment in Enduring Anorexia Nervosa: A Case Series. *European Eating Disorders Review*, *24*(2), 157–163. <https://doi.org/10.1002/erv.2414>
5. McClelland, J., Kekic, M., Bozhilova, N., Nestler, S., Dew, T., Van den Eynde, F., … Schmidt, U. (2016). A Randomised Controlled Trial of Neuronavigated Repetitive Transcranial Magnetic Stimulation (rTMS) in Anorexia Nervosa. *PloS One*, *11*(3), e0148606–e0148606. <https://doi.org/10.1371/journal.pone.0148606>
6. Choudhary, P., Roy, P., and Kumar Kar, S. (2017). Improvement of weight and attitude towards eating behaviour with high frequency rTMS augmentation in anorexia nervosa. *Asian Journal of Psychiatry*, *28*, 160–160. <https://doi.org/10.1016/j.ajp.2017.05.010>
7. Jaššová, K., Albrecht, J., Papežová, H., and Anders, M. (2018). Repetitive Transcranial Magnetic Stimulation (rTMS) Treatment of Depression and Anxiety in a Patient with Anorexia Nervosa. *Medical Science Monitor*, *24*, 5279–5281. <https://doi.org/10.12659/MSM.908250>
8. Woodside, D. B., Colton, P., Lam, E., Dunlop, K., Rzeszutek, J., and Downar, J. (2017). Dorsomedial prefrontal cortex repetitive transcranial magnetic stimulation treatment of posttraumatic stress disorder in eating disorders: An open‐label case series. *The International Journal of Eating Disorders*, *50*(10), 1231–1234. <https://doi.org/10.1002/eat.22764>
9. Dalton, B., Bartholdy, S., McClelland, J., Kekic, M., Rennalls, S. J., Werthmann, J., … Schmidt, U. (2018). Randomised controlled feasibility trial of real versus sham repetitive transcranial magnetic stimulation treatment in adults with severe and enduring anorexia nervosa: the TIARA study. *BMJ Open*, *8*(7), e021531–e021531. <https://doi.org/10.1136/bmjopen-2018-021531>
10. Knyahnytska, Y. O., Blumberger, D. M., Daskalakis, Z. J., Zomorrodi, R., and Kaplan, A. S. (2019). Insula H-coil deep transcranial magnetic stimulation in severe and enduring anorexia nervosa (SE-AN): a pilot study. *Neuropsychiatric Disease and Treatment*, *15*, 2247–2256. <https://doi.org/10.2147/NDT.S207630>
11. Dalton, B., Foerde, K., Bartholdy, S., McClelland, J., Kekic, M., Grycuk, L., … Steinglass, J. E. (2020). The effect of repetitive transcranial magnetic stimulation on food choice‐related self‐control in patients with severe, enduring anorexia nervosa. *The International Journal of Eating Disorders*, *53*(8), 1326–1336. <https://doi.org/10.1002/eat.23267>
12. Muratore AF, Bershad M, Steinglass JE, et al. Use of high-frequency repetitive transcranial magnetic stimulation to probe the neural circuitry of food choice in anorexia nervosa: A proof-of-concept study. *Int J Eat Disord*. 2021;54(11):2031-2036. https://doi.org/ 10.1002/EAT.23597
13. Dunlop, K., Woodside, B., Lam, E., Olmsted, M., Colton, P., Giacobbe, P., and Downar, J. (2015). Increases in frontostriatal connectivity are associated with response to dorsomedial repetitive transcranial magnetic stimulation in refractory binge/purge behaviors. *NeuroImage Clinical*, *8*(C), 611–618. <https://doi.org/10.1016/j.nicl.2015.06.008>

**References (Supplementary Table 1 - TDCS)**

1. Khedr, E.M.; Elfetoh, N.A.; Ali, A.M.; Noamany, M. Anodal transcranial direct current stimulation over the dorsolateral prefrontal cortex improves anorexia nervosa: A pilot study. Restor. Neurol. Neurosci. 2014, 32, 789–797.
2. Strumila, R.; Thiebaut, S.; Jaussent, I.; Seneque, M.; Attal, J.; Courtet, P.; Guillaume, S. Safety and efficacy of transcranial direct current stimulation (tDCS) in the treatment of Anorexia Nervosa. The open-label STAR study. Brain Stimul. 2019, 12, 1325–1327.
3. Costanzo F, Menghini D, Maritato A, et al. New treatment perspectives in adolescents with anorexia nervosa: The efficacy of non-invasive brain-directed treatment. *Front Behav Neurosci*. 2018;12:133. doi:10.3389/FNBEH.2018.00133/BIBTEX
4. Baumann S, Mareš T, Albrecht J, et al. Effects of Transcranial Direct Current Stimulation Treatment for Anorexia Nervosa. *Front Psychiatry*. 2021;12:1626. doi:10.3389/FPSYT.2021.717255/BIBTEX

**References (Supplementary Table 1 - ECT)**

1. Bernstein, I.C. Anorexia Nervosa Treated Successfully with Electroshock Therapy and Subsequently Followed by Pregnancy. Am. J. Psychiatry 1964, 120, 1023–1024. 2. Ferguson, J.M. The use of electroconvulsive therapy in patients with intractable anorexia nervosa. Int. J. Eat. Disord. 1993, 13, 195–201.

3. Hill, R.; Haslett, C.; Kumar, S. Anorexia nervosa in an elderly woman. Aust. N. Z. J. Psychiatry 2001, 35, 246–248.

4. Poutanen, O.; Huuhka, K.; Perko, K. Severe anorexia nervosa, co-occurring major depressive disorder and electroconvulsive therapy as maintenance treatment: A case report. Cases J. 2009, 2, 9362. 5. Andrews, J.T.; Seide, M.; Guarda, A.S.; Redgrave, G.W.; Co ey, D.B. Electroconvulsive therapy in an adolescent with severe major depression and anorexia nervosa. J. Child. Adolesc. Psychopharmacol. 2014, 24, 94–98. 6. Andersen, L.; LaRosa, C.; Gih, D.E. Reexamining the Role of Electroconvulsive Therapy in Anorexia Nervosa in Adolescents. J. ECT 2017, 33, 294–296. 7. Saglam, T.; Aksoy Poyraz, C.; Poyraz, B.C.; Tosun, M. Successful use of electroconvulsive therapy in a patient with anorexia nervosa and severe acute-onset obsessive-compulsive disorder. Int. J. Eat. Disord. 2018, 51, 1026–1028. 8. Pacilio, R.M.; Livingston, R.K.; Gordon, M.R. The Use of Electroconvulsive Therapy in Eating Disorders: A Systematic Literature Review and Case Report. J. ECT 2019, 35, 272–278. 9. Naguy, A.; Al-Tajali, A.; Alamiri, B. An Adolescent Case of Treatment-Refractory Anorexia Nervosa Favorably Responded to Electroconvulsive Therapy. J. ECT 2019, 35, 217–218. 10. Duriez, P.; Maatoug, R.; Verbe, J. Failure of Electroconvulsive Therapy to Improve Anorexia Nervosa in the Absence of Other Psychiatric Comorbidities: A Case Report. J. ECT 2020. 11. Shilton, T.; Enoch-Levy, A.; Giron, Y.; Yaroslavsky, A.; Amiaz, R.; Gothelf, D.; Weizman, A.; Stein, D. A retrospective case series of electroconvulsive therapy in the management of comorbid depression and anorexia nervosa. Int. J. Eat. Disord. 2020, 53, 210–218.

12. Davis, H.K. Anorexia nervosa: Treatment with hypnosis and ECT. Dis. Nerv. Syst. 1961, 22, 627–631.

13. Bernstein, I.C. Anorexia nervosa, 94-year-old woman treated with electroshock. Minn. Med. 1972, 55, 552–553.

14. Bek, R.; Hotujak, L. Clinical characteristics of female patients su ering from anorexia nervosa. Soc. Psihijat. 1996, 24, 159–161.

**References (Supplementary Table 1 - DBS)**

1. Israel, M.; Steiger, H.; Kolivakis, T.; McGregor, L.; Sadikot, A.F. Deep brain stimulation in the subgenual cingulate cortex for an intractable eating disorder. Biol. Psychiatry 2010, 67, e53-e54. 2. Barbier, J.; Gabriels, L.; van Laere, K.; Nuttin, B. Successful anterior capsulotomy in comorbid anorexia nervosa and obsessive-compulsive disorder: Case report. Neurosurgery 2011, 69, E745–E751, discussion E751. 3. McLaughlin, N.C.; Didie, E.R.; Machado, A.G.; Haber, S.N.; Eskandar, E.N.; Greenberg, B.D. Improvements in anorexia symptoms after deep brain stimulation for intractable obsessive-compulsive disorder. Biol. Psychiatry 2013, 73, e29–e31. 4. Wu, H.; Van Dyck-Lippens, P.J.; Santegoeds, R.; van Kuyck, K.; Gabriels, L.; Lin, G.; Pan, G.; Li, Y.; Li, D.; Zhan, S.; et al. Deep-brain stimulation for anorexia nervosa. World Neurosurg. 2013, 80, S29.e1–S29.e10. 5. Wang, J.; Chang, C.; Geng, N.;Wang, X.; Gao, G. Treatment of intractable anorexia nervosa with inactivation of the nucleus accumbens using stereotactic surgery. Stereotact. Funct. Neurosurg. 2013, 91, 364–372. 6. Zhang HW, Li DY, Zhao J, Guan YH, Sun BM, Zuo CT. Metabolic imaging of deep brain stimulation in anorexia nervosa: A 18F-FDG PET/CT study. *Clin Nucl Med*. 2013;38(12):943-948. doi:10.1097/RLU.0000000000000261

7. Lipsman, Woodside, D. B., Giacobbe, P., Hamani, C., Carter, J. C., Norwood, S. J., Sutandar, K., Staab, R., Elias, G., Lyman, C. H., Smith, G. S., and Lozano, A. M. (2013). Subcallosal cingulate deep brain stimulation for treatment-refractory anorexia nervosa: a phase 1 pilot trial. *The Lancet (British Edition)*, *381*(9875), 1361–1370.

8. Hayes, D.J.; Lipsman, N.; Chen, D.Q.;Woodside, D.B.; Davis, K.D.; Lozano, A.M.; Hodaie, M. Subcallosal Cingulate Connectivity in Anorexia Nervosa Patients Differs From Healthy Controls: A Multi-tensor Tractography Study. Brain Stimul. 2015, 8, 758–768. 9. Lipsman, N.; Lam, E.; Volpini, M.; Sutandar, K.; Twose, R.; Giacobbe, P.; Sodums, D.J.; Smith, G.S.; Woodside, D.B.; Lozano, A.M. Deep brain stimulation of the subcallosal cingulate for treatment-refractory anorexia nervosa: 1 year follow-up of an open-label trial. Lancet Psychiatry 2017, 4, 285–294. 10. Blomstedt, P.; Naesstrom, M.; Bodlund, O. Deep brain stimulation in the bed nucleus of the stria terminalis and medial forebrain bundle in a patient with major depressive disorder and anorexia nervosa. Clin. Case Rep. 2017, 5, 679–684. 11. Manuelli, M.; Franzini, A.; Galentino, R.; Bidone, R.; Dell’Osso, B.; Porta, M.; Servello, D.; Cena, H. Changes in eating behavior after deep brain stimulation for anorexia nervosa. A case study. Eat. Weight Disord. 2019. 12. Liu, W.; Zhan, S.; Li, D.; Lin, Z.; Zhang, C.; Wang, T.; Pan, S.; Zhang, J.; Cao, C.; Jin, H.; et al. Deep brain stimulation of the nucleus accumbens for treatment-refractory anorexia nervosa: A long-term follow-up study. Brain Stimul. 2020, 13, 643–649.

13. Arroteia IF, Husch A, Baniasadi M, Hertel F. Impressive weight gain after deep brain stimulation of nucleus accumbens in treatment-resistant bulimic anorexia nervosa. *BMJ Case Reports CP*. 2020;13(11):e239316. doi:10.1136/BCR-2020-239316

14. Lin Z, Zhang C, Zhang Y, et al. Deep brain stimulation telemedicine programming during the COVID-19 pandemic: treatment of patients with psychiatric disorders. *Neurosurg Focus*. 2020;49(6):E11. doi:10.3171/2020.9.FOCUS20666

15. De Vloo P, Lam E, Elias GJB, et al. Long-term follow-up of deep brain stimulation for anorexia nervosa. *J Neurol Neurosurg Psychiatry*. 2021;92(10):1135-1136. doi:10.1136/JNNP-2020-325711

16. Oudijn MS, Mocking RJT, Wijnker RR, et al. Deep brain stimulation of the ventral anterior limb of the capsula interna in patients with treatment-refractory anorexia nervosa. *Brain Stimul*. 2021;14(6):1528-1530. doi:10.1016/j.brs.2021.10.387

17. Scaife JC, Eraifej J, Green AL, Petric B, Aziz TZ, Park RJ. Deep Brain Stimulation of the Nucleus Accumbens in Severe Enduring Anorexia Nervosa: A Pilot Study. *Front Behav Neurosci*. 2022;16:76. doi:10.3389/FNBEH.2022.842184/BIBTEX

18. Villalba Martinez, G.; Justicia, A.; Salgado, P.; Gines, J.M.; Guardiola, R.; Cedron, C.; Polo, M.; Delgado-Martinez, I.; Medrano, S.; Manero, R.M.; et al. A Randomized Trial of Deep Brain Stimulation to the Subcallosal Cingulate and Nucleus Accumbens in Patients with Treatment-Refractory, Chronic, and Severe Anorexia Nervosa: Initial Results at 6 Months of Follow Up. J. Clin. Med. 2020, 9, 1946.
